# STRAP as a New Therapeutic Target for Poor Prognosis of Pancreatic Ductal Adenocarcinoma Patients Mainly Caused by *TP53* Mutation

**DOI:** 10.3389/fonc.2020.594224

**Published:** 2020-09-29

**Authors:** Shanshan Hu, Xiao Chen, Xiangxiang Xu, Chenlei Zheng, Wenqian Huang, Yi Zhou, Percy David Papa Akuetteh, Hongbao Yang, Keqing Shi, Bicheng Chen, Qiyu Zhang

**Affiliations:** Key Laboratory of Diagnosis and Treatment of Severe Hepato-Pancreatic Diseases of Zhejiang Province, The First Affiliated Hospital of Wenzhou Medical University, Wenzhou, China

**Keywords:** pancreatic ductal adenocarcinoma, *TP53*, prognosis, snoRNA, STRAP

## Abstract

Pancreatic ductal adenocarcinoma (PDAC) has a high mortality rate and poor prognosis. *KRAS*, *TP53*, *CDKN2A*, and *SMAD4* are driver genes of PDAC and 30–75% patients have mutations in at least two of these four genes. Herein, we analyzed the relationship between these genes and prognosis of 762 patients in the absence of coexisting mutations, using data from three independent public datasets. Interestingly, we found that compared with mutations in other driver genes, *TP53* mutation plays a significant role in leading to poor prognosis of PDAC. Additionally, we found that snoRNA-mediated rRNA maturation was responsible for the progression of cancer in PDAC patients with *TP53* mutations. Inhibition of STRAP, which regulates the localization of SMN complexes and further affects the assembly of snoRNP, can effectively reduce maturation of rRNA and significantly suppress progression of *TP53*-mutant or low p53 expression pancreatic cancer cells *in vitro* and *in vivo*. Our study highlighted the actual contribution rate of driver genes to patient prognosis, enriching traditional understanding of the relationship between these genes and PDAC. We also provided a possible mechanism and a new target to combat progression of *TP53*-mutant PDAC patients.

## Introduction

Pancreatic ductal adenocarcinoma (PDAC) is one of the deadliest human malignancies (1), with >95% mortality rate and a 5-year survival rate of less than 9% (2). It is known as the “king of cancer” due to its high degree of malignancy and currently the fourth leading cause of cancer-related deaths in the United States (2), and is expected to become the second within the next decade (3). Surgical resection is the only curative treatment for PDAC; however, this tumor is difficult to detect and quickly spreads locally or metastasizes to distant organs by the time of initial diagnosis. Therefore, less than 20% of patients have a chance of resection (4). Furthermore, most patients who undergo pancreatic resection experience local or systemic recurrences, with a median post-resection survival rate of less than 20 months (5, 6). Therefore, finding the underlying mechanisms that influence the prognosis of PDAC is an urgent need requiring the exploration of novel adjuvant therapeutic strategies to improve the survival rate of patients.

Studies have shown that the occurrence of PDAC is caused by genetic mutations (7, 8). In recent years, with the development of next-generation sequencing technology, alterations in hundreds of genes related to axon guidance, DNA damage repair, chromatin remodelers, cell cycle regulation, and focal amplifications in druggable genes have been identified by whole genome, whole exome, and targeted deep sequencing in a large number of PDAC patients (9–11). *KRAS*, *TP53*, *CDKN2A*, and *SMAD4*, referred to as “driver genes,” are the most frequently mutated genes and are well recognized as a contributing factor to pancreatic carcinogenesis (12, 13). Mutations in *KRAS* are present in more than 90% of patients (14) and are known to be related to the initiation of PDAC (15). Inactivating mutations of *TP53*, *CDKN2A*, and *SMAD4* occurred in 15–80% of PDAC patients and 30–75% had mutations in at least two of the four genes (16–18). Several studies (19–21) explored the relationship between driver genes and prognosis and largely found that these genes were associated with disease prognosis. Further, other studies (22, 23) showed that the higher the number of mutations occurring in these driver genes, the worse the prognosis, especially in patient with mutations in more than three genes. However, these studies did not take into account the possible effects of coexistence of mutations in the driver genes.

To explore the actual contribution rate of the four-driver genes to this disease, we analyzed the influence of mutation in a single gene on the prognosis of patients based on extensive sample sequencing data derived from public databases. This study also explored the possible mechanism affecting prognosis of PDAC and then investigated potential novel adjuvant therapeutic targets *in vitro* and *in vivo*.

## Materials and Methods

### Patient Material Acquisition and Extraction

Data for clinical parameters, somatic mutations, and gene expression of PDAC patients were downloaded from The Cancer Genome Atlas (TCGA) Portal^1^ and two other independent studies whose data were stored in the International Cancer Genome Consortium (ICGC) data portal^2^.

### Mutation Annotation and Filtering

All mutations obtained from public datasets were subjected to re-annotation by ANNOVAR (24) as described in our previous studies (25, 26), including cytoband, gene region, functional effect, and amino-acid change. Then, we screened mutations in the exon region because these mutations might affect the function of the protein.

### Survival Analysis

Multivariate Cox hazard regression was used to assess the impact of some prognostic factors. Then, we used the anova() function to estimate the significance of each variable. Median survival time and cumulative survival curves were determined by the Kaplan–Meier method and differences between/among the groups were analyzed using the log-rank test. *P* < 0.05 was considered statistically significant.

### Differential Expression Analysis of Genes

Only genes with a normalized expression value more than 0 in over 20% of the samples were considered to be expressed. Differentially expressed genes (DEGs) of different prognosis subtypes were determined with Student’s two-tailed *t*-test. Since genes with expression levels that were too low reduced statistical credibility, we first excluded genes with expression levels below 5 in both of the groups used for comparison. Genes with a *P* ≤ 0.05 and | log2FoldChange| ≥ 1 were defined as differential genes. Simultaneously, RankCompV2 (27), a rank-based algorithm, was used for differential expression analysis and utilized to calculate DEGs with default parameters. This method was not affected by the level of gene expression.

### Functional Enrichment Analysis

To identify enriched pathways and gene ontologies of gene sets, we performed enrichment analysis using the R package ClusterProfiler. For the pathway analysis, we used the pathway annotations package ReactomePA provided by Reactome Pathway Database. GO gene set collections were obtained from GO.db package. We performed Fisher’s exact test and permutation test to calculate *P* and OR values for enrichment analysis of the family genes or cluster genes. The permutation test was based on random sampling, as in our previous study (26). Specifically, we calculated the *P* by comparing the number of differential genes in this family/cluster to the number of genes from the family/cluster of 1,000,000 simulated datasets. Each simulated dataset included the same number of total DEGs by random sampling.

### Cell Lines

Mutant background of the pancreatic cancer cell lines was queried by Cancer Cell Line Encyclopedia (CCLE)^3^.

PANC-1, Patu-8988, and PANC-0327 cells were purchased from the American Type Culture Collection (Manassas, VA, United States). KP4 cell line was obtained from the Riken BioResource Center Cell Bank (Ibaraki, Japan). All the cell lines were cultured in either DMEM or RPMI-1640 media supplemented with 10% fetal bovine serum, and were free of mycoplasmas and authenticated by polymorphic short tandem repeat loci before use.

Cell lines stably overexpressing human p53 in *TP53*-mutant cells or p53-knockdown in *TP53* non-mutant cells were generated by infecting cells with lentiviruses expressing p53 or p53 shRNA (MOI = 10; GeneChem Co. Ltd., Shanghai, China), respectively. STRAP-knockdown cells were generated by infecting cells with lentiviruses expressing two specific STRAP shRNAs (MOI = 10; GeneChem Co. Ltd.). Cells infected with lentiviruses expressing control empty vector or shRNA were used as controls. We selected successfully infected cells with puromycin (1 μg/ml) for 7 days.

### Western Blot Analysis

Western blot analysis was performed as described previously (28, 29). The following commercially available antibodies were used in this study: GAPDH (Cell Signaling Technology, Shanghai, China; catalog no. 2118), p53 (ProteinTech, Wuhan, China; catalog no. 10442-1-AP) and STRAP (ProteinTech; catalog no. 18277-1-AP).

### qPCR

Total RNA was isolated using TRIzol reagent (Life Technologies, Shanghai, China) and reverse-transcribed using the M-MLV reverse transcription kit (Promega, Madison, WI, United States). qPCR was carried out in an ABI 7500 Fast instrument (Life Technologies) using the SYBR Premix Ex Taq kit (TaKaRa, Dalian, China).

### Ribosomal RNA Processing Analyses

We performed qPCR to evaluate rRNA processing. Gene-specific primers of 18S and 28S rRNA (Supplementary Table 1) and the calculation method for the fraction of unprocessed rRNA were determined as described previously by Cao (30). Specifically, the unprocessed rate of 18S rRNA was the averages of primer pairs 4/3 (unprocessed) over 2/1 (total) and primer pairs 6/5 (unprocessed) over 2/1 (total), and that for 28S rRNA was the averages of primer pairs d/c (unprocessed) over b/a (total) and primer pairs f/e (unprocessed) over b/a (total).

### Cell Proliferation, Migration, and Invasion Assays

Lentivirus-transfected pancreatic cancer cells were plated into 96-well plates at a density of 3 × 10^3^ cells per well to test cell proliferation. The Cell Counting Kit-8 (CCK-8) reagent (Dojindo, Kyushu Island, Japan) was used to detect cell viability every 24 h for 3 days. The OD value (450 nm) was recorded to generate a cell proliferation curve.

Wound-healing assays were used to assess the migration ability of cells. Transfected cells were seeded into 12-well plates and then cultured for 24 h until 95% confluence. The confluent monolayer in each well was created using a 1,000 μl pipette tip and cultured for 48 h. Cells were photographed at 0, 24, and 48 h under a Nikon Eclipse TE2000-U Inverted Microscope (Nikon, Tokyo, Japan).

For the invasion assay, 2 × 10^4^ cells per well were plated into the upper chamber of a 24-well Transwell chamber (Corning, NY, United States) and coated with Matrigel and serum-free medium. Then, 500 μl complete medium with 10% FBS was added into the lower chamber. Cell migration through the Matrigel substrate was assessed after 24 h by fixing it in 4% paraformaldehyde, staining with 1% crystal violet (Sigma), and counting the migrated cells by selecting five fields at random under a light microscope.

### Animal Studies

All animal studies and procedures were approved by the Institutional Animal Care and Use Committee of Wenzhou Medical University. Tumor xenografts were generated by adding 5 × 10^6^ Patu-8988 cells with p53 overexpression (LV-pP53) or control empty vector (LV-Con) and 5 × 10^6^ KP4 cells with p53-knockdown (LV-shP53) or control shRNA (LV-shCon) to 100 μl PBS, and then subcutaneously injected into each flank of 6-week-old female athymic BALB/c nude mice. When the volume of the tumors was about 100 mm^3^, mice were randomly assigned to two groups (5 mice/per group) and then received an intratumoral injection of shSTRAP-1 or shCon at a titer of 10^7^ TU in 10 μl PBS every 3 days, which was repeated three times. The volume of tumors was calculated with the following formula: V = (Width^2^ × Length)/2. Mice were sacrificed 35 days following tumor injection. The investigator was not blinded to group allocation during the experiment but was blinded when assessing the xenograft tumor volumes following euthanasia of the mice.

## Results

### Data Collection

In total, we retrieved detailed clinical information from 923 PDAC patients, which included 784 somatic mutations, and 279 RNA sequences from three independent PDAC-related studies, including TCGA, and two other independent studies stored in ICGC (PACA-AU, PACA-CA) (Supplementary Table 2). There were 762 PDAC samples with both survival information and somatic mutation data. Patients of TCGA, PACA-AU, and PACA-CA were from the United States, Australia, and Canada, respectively. Data utilized from all three countries included 154, 461, and 308 follow-up survival data, 133, 391, and 260 somatic mutation information, and 142, 91, and 46 RNA sequence data, respectively.

### Multivariate Analysis of the Clinical Parameters Regarding the Prognosis of Patients With PDAC

Due to the lack of detailed clinical data, we only assessed the impact of some parameters on prognosis (Supplementary Table 3). Using multivariate Cox analysis, we found no difference in survival rates among patients in the three databases (*P* = 0.58). Further, we analyzed the effects of gender and age on the prognosis of patients and found no difference. However, the number of mutations in driver genes had a significant effect on the prognosis of patients (*P* = 0.0028), which is consistent with previous reports (19–21).

Next, we analyzed the mutation frequency of the driver genes in the patients and found that it was consistent with previous results: more than 90% (90.43%) of patients had *KRAS* mutations. Patients carrying *TP53*, *SMAD4*, and *CDKN2A* mutations were 69.13, 23.21, and 20.66%, respectively. Moreover, nearly 75% (74.74%) of patients were carrying more than two mutations at the same time. Among them, 98.63% of patients had *KRAS* mutations (Supplementary Table 4).

### Analysis of Prognosis in Patients With Mutations in Driver Genes

**FIGURE 1:**
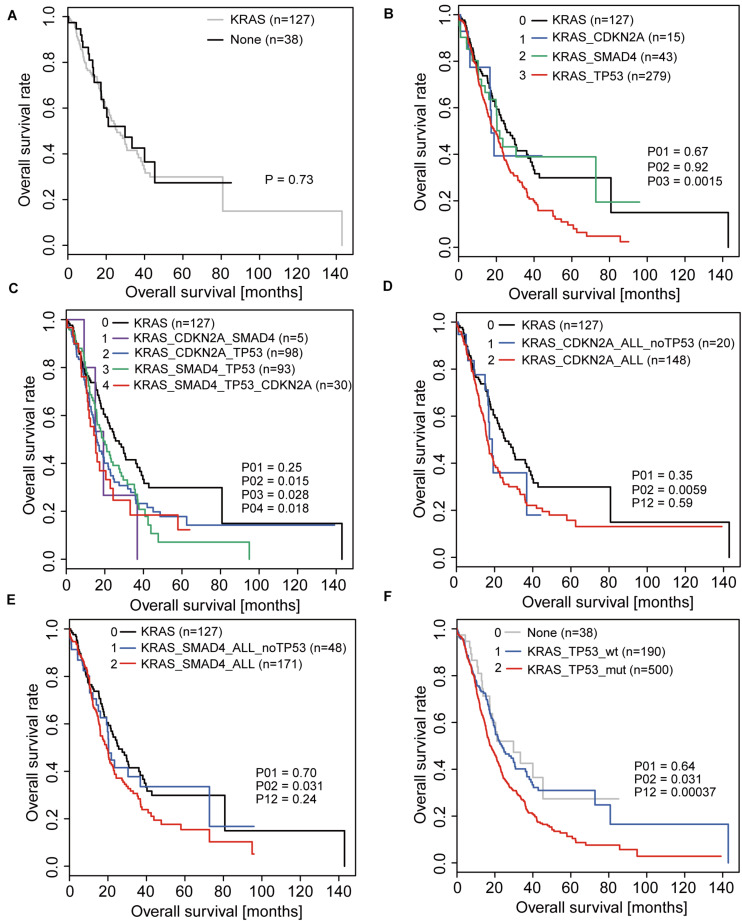
Overall survival (OS) analyses in PDAC patients correlated with driver gene mutations. **(A)** OS of patients with *KRAS* mutations only and without any driver gene mutations. **(B)** OS of patients with mutations in only one driver gene based on the activation of *KRAS*. **(C)** OS of patients with coexistence of mutations in driver genes based on the activation of *KRAS*. **(D,E)** OS of patients with *CDKN2A* mutations **(D)** or *SMAD4* mutations **(E)** when considering and not considering *TP53* mutations. **(F)** OS of patients with and without *TP53* mutations.

Since the *KRAS* mutation is present in almost all patients and is the initiator of the disease, we analyzed the prognosis of patients with only *KRAS* mutations and those without any driver gene mutations and found no difference between the two groups (Figure 1A). Therefore, when considering the contribution of mutations in the other three driver genes to prognosis, activation of *KRAS* was used as the basis; hence we used it as the control group.

When analyzing the effects of *TP53*, *SMAD4*, and *CDKN2A* mutations on the prognosis of patients, we first analyzed the overall survival of patients with mutations in only one of the three driver genes based on the activation of *KRAS* and found that only patients with *TP53* mutations were significantly different from the control group (Figure 1B). Under conditions of coexistence of mutations in two or three driver genes, we also found that only patients with *TP53* mutations simultaneously had a significant difference in prognosis compared to the control group (Figure 1C). This suggests that *TP53* may play a significant role in affecting patient prognosis.

To further confirm whether the influence of other driver gene mutations on prognosis was due to the coexistence of *TP53* mutations, we re-analyzed the relationship between *CDKN2A* and *SMAD4* mutations in relation to prognosis. Consistent with the original conclusion, we found that the prognosis of patients with *CDKN2A* or *SMAD4* mutations was significantly worse than that of patients without mutations when the *TP53* mutation status was not considered (Figures 1D,E). However, when patients with *TP53* mutations were excluded, the prognosis between the two groups of patients exhibited no significant difference (Figures 1D,E).

In summary, the above results indicated that *TP53* is the real key factor leading to poor prognosis. The prognostic analysis revealed that the prognosis of patients with *TP53* mutations was significantly reduced compared to that of patients without *TP53* mutation after *KRAS* activation (Figure 1F).

### *TP53* Affects the Progress of Pancreatic Cancer Cell Lines *in vitro* and *in vivo*

**FIGURE 2:**
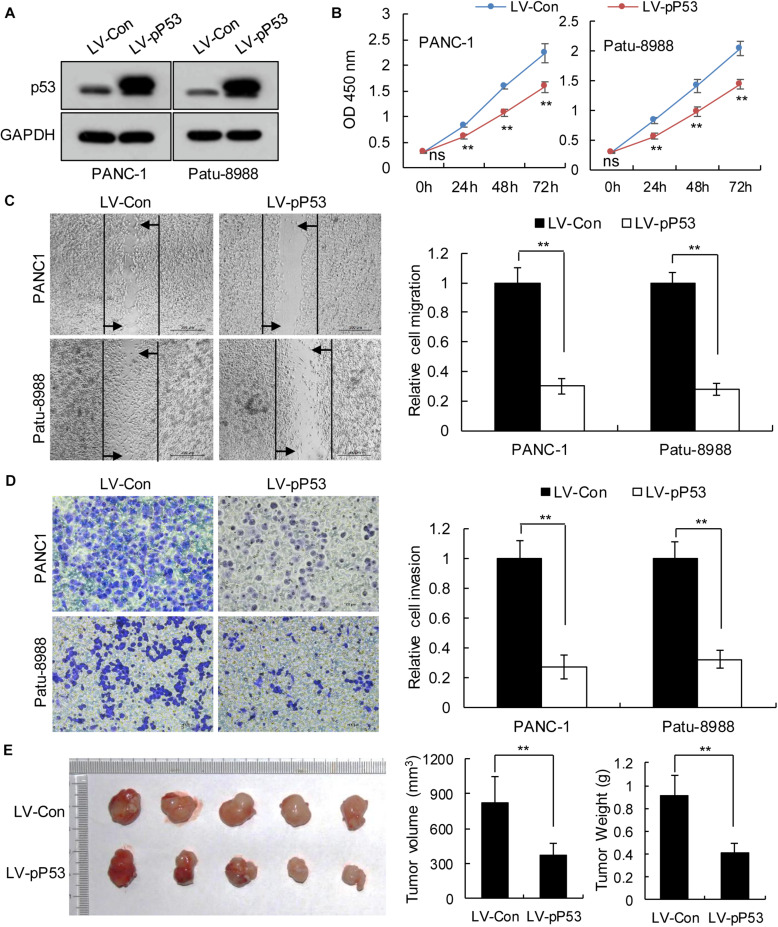
Overexpression of p53 in *TP53*-mutant pancreatic cancer cells suppressed cancer progression *in vitro* and *in vivo*. PANC-1 and Patu-8988 cells were infected with control or over-expressing *TP53* lentiviruses. **(A)** p53 expression was analyzed by western blotting. **(B)** The cell proliferation assay was performed at the indicated time points. **(C)** Representative micrographs of cell migration assays at 48 h (left) and quantification results (right). **(D)** Representative micrographs of cell invasion assays (left) and quantification results (right). Data in panels **(B–D)** are shown as the mean ± SEM of 3 independent experiments. **(E)** Representative images, volumes and weights of subcutaneous xenografts of Patu-8988 cells with overexpressing p53 or control. Data represent means ± SEM for 5 mice per group. **P < 0.01.

**FIGURE 3:**
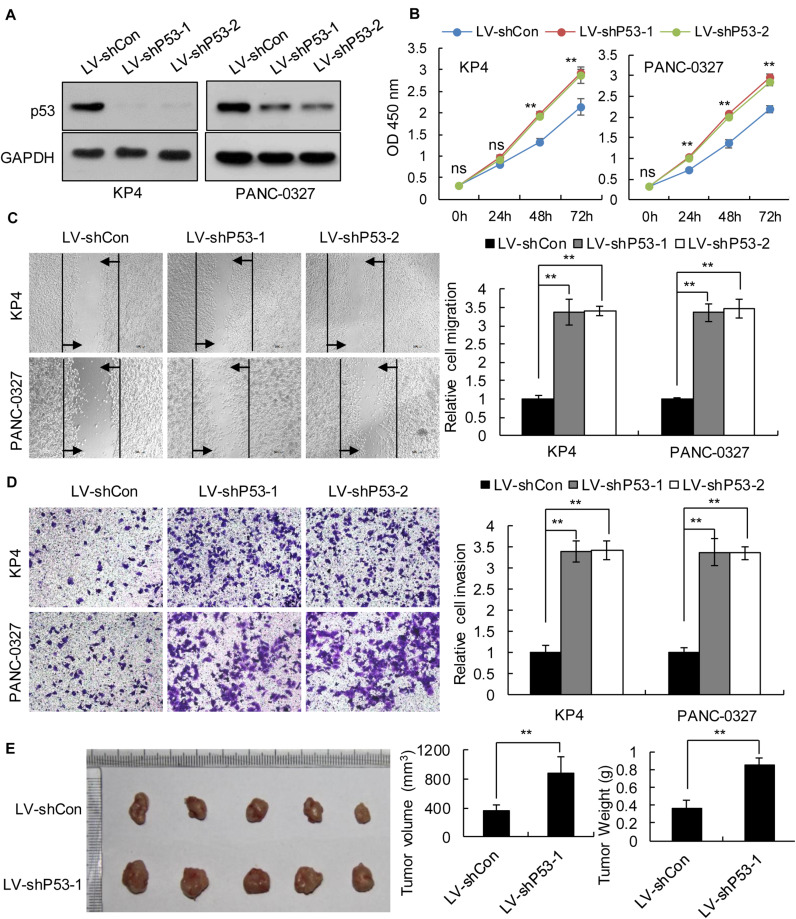
Knockdown of p53 in *TP53* non-mutant pancreatic cancer cells promoted cancer progression *in vitro* and *in vivo*. KP4 and PANC-0327 cells were infected with control or p53-knockdown lentiviruses. **(A)** p53 expression was analyzed by western blotting. **(B)** The cell proliferation assay was performed at the indicated time points. **(C)** Representative micrographs of cell migration assays at 48 h (left) and quantification results (right). **(D)** Representative micrographs of cell invasion assays (left) and quantification results (right). Data in panels **(B–D)** are shown as the mean ± SEM of 3 independent experiments. **(E)** Representative images, volumes and weights of subcutaneous xenografts of KP4 cells with p53 knockdown or control. Data represent means ± SEM for 5 mice per group. **P < 0.01.

We selected pancreatic cancer cell lines PANC1 and Patu-8988 with both *KRAS* and *TP53* mutations and KP-4 and PANC-0327 with only *KRAS* mutations to verify the dependence of pancreatic cancer survival on *TP53* (Supplementary Table 5). CCK8 proliferation assay results demonstrated that overexpression of *TP53* (LV-pP53) in PANC1 and Patu-8988 displayed a significant decrease in cell proliferation compared with that in the control group (LV-Con) (Figures 2A,B). Wound-healing assays indicated that the migration distance of the LV-pP53 group was shorter than that of the control group (Figure 2C). In parallel, the results of the Transwell invasion assay showed that the invasion ability of LV-pP53 was lower than that of the control group (Figure 2D). Similarly, we also found that overexpressing p53 could effectively suppress xenograft tumor growth (Figure 2E). However, compared with those in the control group (LV-shCon), the proliferation, migration, invasion, and xenograft tumor growth of p53-knockdown in KP-4 and PANC-0327 (LV-shP53) were promoted (Figure 3).

### Analysis of Differentially Expressed Genes and Their Functional Pathways

In order to find out the mechanism by which *TP53* mutation affects prognosis, we divided patients from public sources into two groups based on *TP53* mutation status: *TP53*_mut and *TP53*_wt. Then, we analyzed DEGs by Student t-test and RankCompV2. A total of 90 DEGs were identified by Student’s t-test, including 73 upregulated and 17 downregulated. RankCompV2 also found 90 DEGs, with 60 upregulated and 30 downregulated (Supplementary Table 6). We performed GO and pathway enrichment analyses to further investigate functional pathways associated with the DEGs. Results showed that genes were enriched in several biological processes and pathways that are known to be associated with nucleosome assembly and the transcriptional regulation of genes, such as chromatin assembly (GO: 0031497), DNA packaging (GO: 0006323), chromatin silencing (GO: 0006342) and RNA Polymerase I Promoter Opening (R-HSA-73728), HDACs deacetylate histones (R-HSA-3214815), and DNA methylation (R-HSA-5334118) (Supplementary Figure 1 and Supplementary Tables 7, 8). More importantly, we noted that the functional pathways were involved in the regulation of rDNA (chromatin silencing at rDNA; GO: 0000183) and rRNA expression (SIRT1 negatively regulated rRNA expression; R-HSA-427359, NoRC negatively regulated rRNA expression; R-HSA-427413, B-WICH complex positively regulated rRNA expression; R-HSA-5250924) as well as the high enrichment of Cajal bodies RNAs and the snoRNA family genes (Table 1).

**TABLE 1:** Functional enrichment analysis of snoRNA family genes and related cluster genes.

	Background	T test	Fisher’s exact test		Permutation test		Background	RankCompV2	Fisher’s exact test		Permutation test	
Total	11789	90	*P*	OR	*P*	OR	16859	90	*P*	OR	*P*	OR
box H/ACA snoRNAs	28	10	1.48e-13	52.39 (21.94–115.58)	<1.00e-06	48.45 (48.25–48.66)	48	4	1.66e-04	16.28 (4.17–45.88)	1.19e-04	15.62 (15.56–15.68)
box C/D snoRNAs	5	2	1.16e-03	53.46 (5.02–330.76)	5.48e-04	52.27 (51.76–52.80)	7	0	1.00	0.00 (0.00–131.85)	1.00	0.00 (0.00–0.00)
Total snoRNA	33	12	3.93e-16	54.69 (24.75–113.52)	1.75e-03	13.66 (13.61–13.71)	55	4	2.71e-04	14.20 (3.66–39.78)	1.75e-04	13.66 (13.61–13.71)
Cajal bodies RNAs	7	2	1.97e-03	38.17 (3.82–205.03)	1.128e-03	37.62 (37.31–37.94)	13	3	7.71e-05	44.60 (8.01–166.48)	2.80e-05	43.19 (42.87–43.51)

### *TP53* Affects the Maturation of Ribosomal RNAs

**FIGURE 4:**
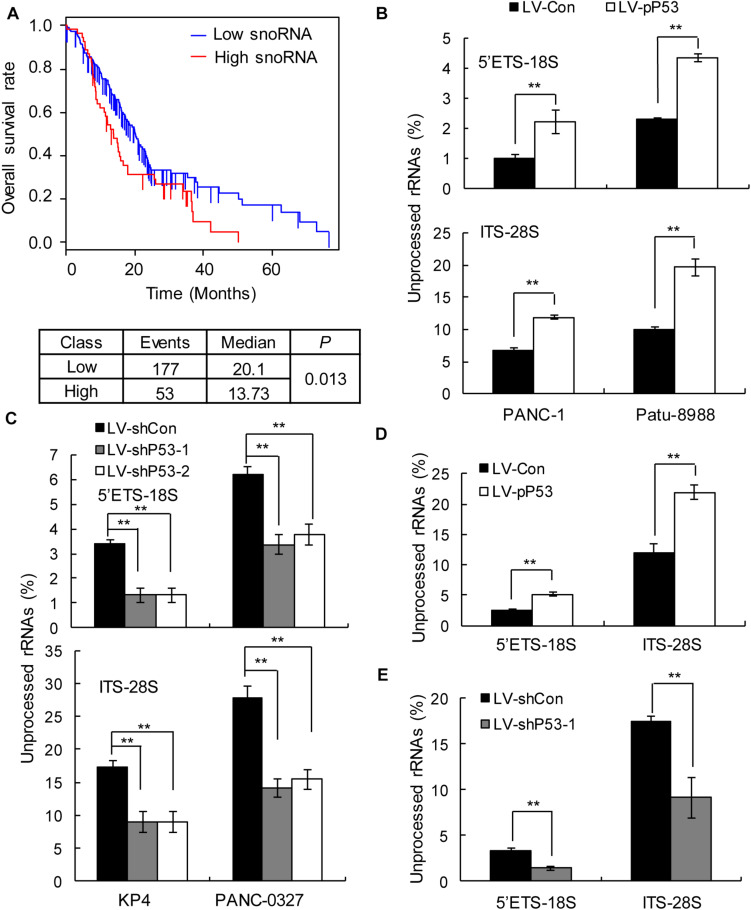
p53 expression is associated with snoRNA-mediated ribosome maturation. **(A)** Kaplan–Meier survival curves for PDAC patients according to the snoRNA family genes in tumor tissues, and significance was calculated using the log-rank test. **(B,D)** Overexpression of p53 in *TP53*-mutant cells *in vitro* **(B)** and *in vivo* **(D)** promotes rRNA processing by detecting 5’ETS-18S and ITS–28S using quantitative reverse transcriptase PCR assays in PANC-1 (left) and Patu-8988 cells (right). **(C,E)** Knockdown p53 in *TP53* non-mutant cells *in vitro* **(C)** and *in vivo* **(E)** suppresses rRNA processing by detecting 5’ETS-18S and ITS–28S using quantitative reverse transcriptase PCR assays in KP4 (left) and PANC-0327 cells (right). The data are shown as the mean ± SEM of 3 independent experiments. **P < 0.01.

In humans, snoRNAs are primarily responsible for the modification and maturation of ribosomal RNAs (rRNAs) (31). Global control of protein synthesis is crucial for cancer development and progression, as highly proliferating cancer cells require increased protein synthesis (32); therefore, more rRNAs may be needed to participate in protein synthesis. Thus, we hypothesized that snoRNA-mediated rRNA maturation might be a cause of cancer progression in patients with *TP53* mutations. The prognostic analysis showed that upregulated snoRNA gene expression was significantly associated with poor prognosis (Figure 4A). qPCR analysis showed that the proportion of mature 18S and 28S rRNA was significantly decreased in the p53 overexpressing PANC1 and Patu-8988 *in vitro* and *in vivo* (Figures 4B,D), whereas p53 knockdown in KP-4 and PANC-0327 promoted the maturation of 18S and 28S rRNA *in vitro* and *in vivo* (Figures 4C,E).

### Knockdown of STRAP Effectively Blocks the Progression of Pancreatic Cancer Cells With Low p53 Expression *in vitro* and *in vivo*

STRAP, also known as UNRIP, is a serine/threonine kinase receptor-associated protein. Krastev et al. (33) found that STRAP affected the localization of SMN complex in a p53-independent manner, which in turn affected the assembly of snoRNP. Inhibition of STRAP could effectively reduce the proliferation and migration of *TP53*-mutant colon cancer cells without affecting the growth of *TP53* non-mutated cancer cells (33). Our prognostic analysis of public data showed that downregulated STRAP significantly improved the prognosis of patients with PDAC (Supplementary Figure 2A), with the effect being better in the *TP53* mutant state than in the non-mutated state (Supplementary Figures 2B,C).

**FIGURE 5:**
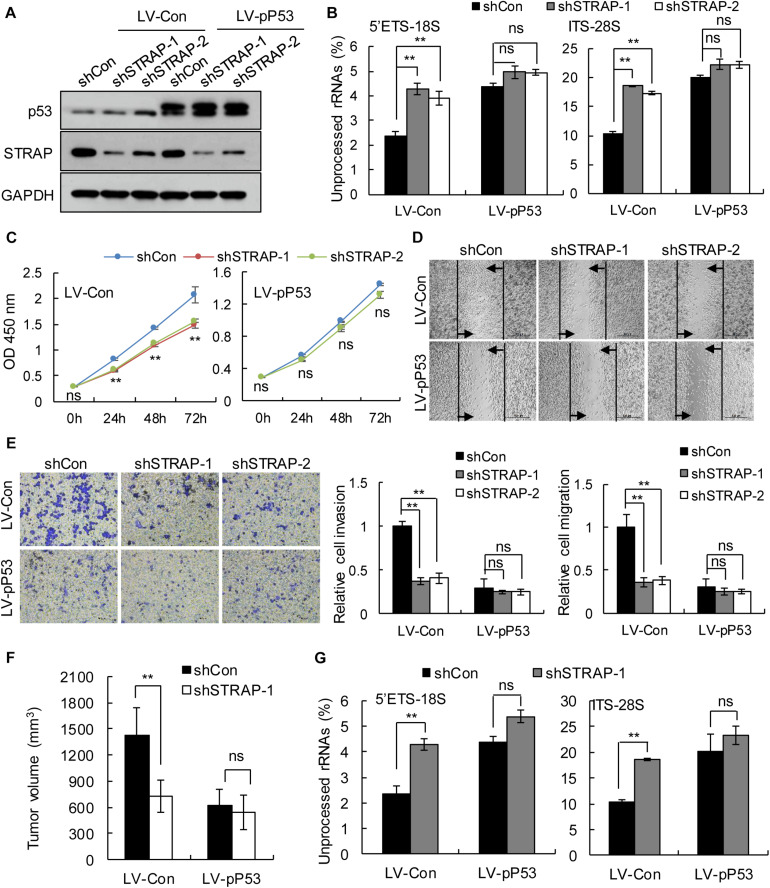
STRAP knockdown suppressed progression of *TP53*-mutant Patu-8988 cells by inhibiting snoRNA-mediated rRNA maturation. Patu-8988 cells with p53 overexpression or control vector were infected with control or STRAP-knockdown lentiviruses. **(A)** p53 and STRAP expression were analyzed by western blotting. **(B,G)** rRNA processing *in vitro* **(B)** and *in vivo* **(G)** by detecting 5’ETS-18S and ITS–28S using quantitative reverse transcriptase PCR assays. The data are shown as the mean ± SEM of 3 independent experiments. **(C)** The cell proliferation assay was performed at the indicated time points. **(D)** Representative micrographs of cell migration assays at 48 h (top) and quantification results (bottom). **(E)** Representative micrographs of cell invasion assays (left) and quantification results (right). Data in panels **(C–E)** are shown as the mean ± SEM of 3 independent experiments. **(F)** Representative volumes of subcutaneous xenografts of Patu-8988 cells with overexpressing p53 or control injected intratumorally with control or STRAP- knockdown lentivirus. Data represent means ± SEM for 5 mice per group. **P < 0.01.

**FIGURE 6:**
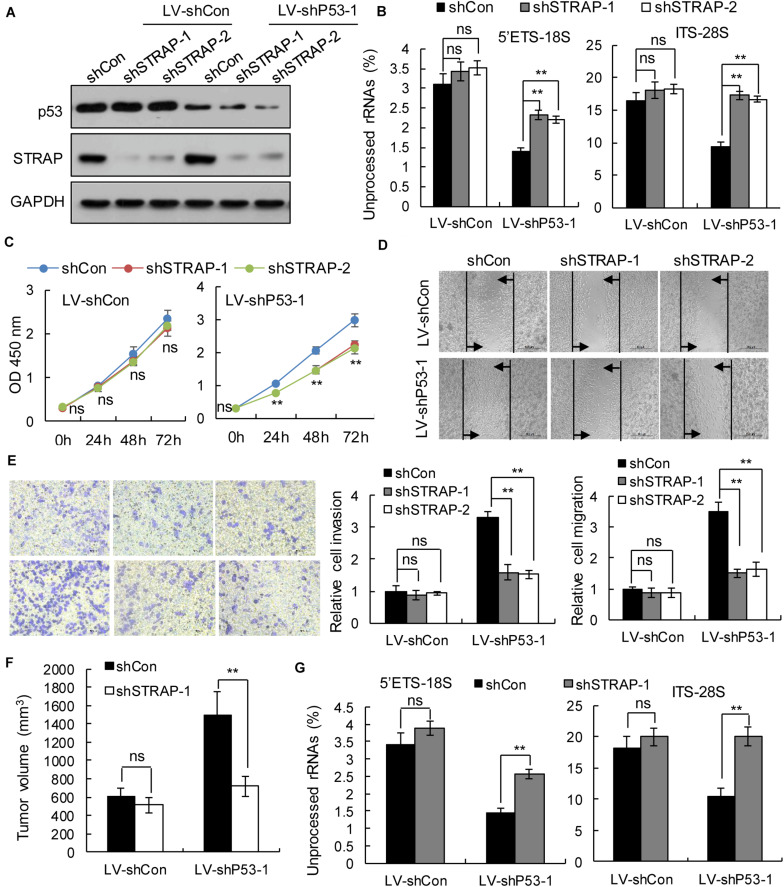
STRAP knockdown suppressed progression of p53-knockdown KP4 cells by inhibiting snoRNA-mediated rRNA maturation. KP4 cells with p53 knockdown or control vector were infected with control or STRAP-knockdown lentiviruses. **(A)** p53 and STRAP expression were analyzed by western blotting. **(B,G)** rRNA processing *in vitro* **(B)** and *in vivo* **(G)** by detecting 5’ETS-18S and ITS–28S using quantitative reverse transcriptase PCR assays. The data are shown as the mean ± SEM of 3 independent experiments. **(C)** The cell proliferation assay was performed at the indicated time points. **(D)** Representative micrographs of cell migration assays at 48 h (top) and quantification results (bottom). **(E)** Representative micrographs of cell invasion assays (left) and quantification results (right). Data in panels **(C–E)** are shown as the mean ± SEM of 3 independent experiments. **(F)** Representative volumes of subcutaneous xenografts of KP4 cells with p53 knockdown or control injected intratumorally with control or STRAP- knockdown lentivirus. Data represent means ± SEM for 5 mice per group. **P < 0.01.

To verify whether inhibition of STRAP was effective against *TP53*-mutant pancreatic cancer cells by inhibiting snoRNA-mediated rRNA maturation, we successfully constructed STRAP-interfering stable cell lines based on p53 overexpression (Figure 5A and Supplementary Figure 3A) or p53-knockdown (Figure 6A and Supplementary Figure 4A). Both *in vitro and in vivo*, knockdown of STRAP in the *TP53* mutant state (Figures 5B–G and Supplementary Figures 3B–E) or p53-knockdown (Figures 6B–G and Supplementary Figures 4B–E) did indeed inhibit rRNA maturation and could effectively inhibit the development of cancer *in vitro* and *in vivo*, but there was no significant effect on the high p53 expression cell lines.

## Discussion

As the “king of cancer,” PDAC has a high mortality rate and poor prognosis (2). Therefore, it is of great significance to search for the key factors that affect the prognosis of PDAC patients and effective adjuvant treatment measures for clinical treatment of PDAC and increase the prognostic survival rate of patients. It is known that *KRAS*, *TP53*, *CDKN2A* and *SMAD4* play an important role in the development of PDAC (12, 13), and are significantly associated with the prognostic survival of patients (19–23). However, mutations in at least two of these four driver genes are present in 30–75% of patients with PDAC (16–18). Is there a bias in the contribution of driver genes to patient survival? Will the relationship between a driver gene and patient prognosis be affected by mutations in other driver genes? This has not been noticed in previous research.

As the most frequent mutated genes in PDAC, *KRAS*, *TP53*, *CDKN2A* and *SMAD4* have been well explored in many studies regarding their relationship with the prognosis of PDAC patients (19–23). Herein, we analyzed data from 762 patients with PDAC and explored the actual contribution rate of four driver genes to prognosis in the absence of coexisting mutations by combining multiple statistical and bioinformatics methods. This study differs from previous studies in that it considers the large number of coexisting mutations of the four driver genes in patients with PDAC, which may partially obscure the true contribution of each gene to patient prognostic survival rate. Interestingly, we found that compared with patients with other driver gene mutations, only patients with *TP53* mutations simultaneously had a significantly lower prognosis than patients in the control group. Additionally, we found that the relationship between other driver gene mutations and prognosis will be affected by the existence of *TP53* mutations. This reminds us that when studying the relationship between other driver genes and PDAC in the future, it is necessary to consider that the coexistence of *TP53* mutations may have an impact on the results. Through *in vitro* and *in vivo* experiments, we also verified the necessity of p53 for the growth of pancreatic cancer. This finding highlighted the actual contribution rate and enriched the traditional understanding of the relationship between these genes and prognosis. However, the results were only based on the univariate analysis of driver gene mutation, other clinical parameters such as age and gender might also have some influence on the result. This requires in-depth research to enrich our conclusions in the future. In this study, we also explored the possible mechanism of p53 affecting patients prognosis. We found that compared with *TP53* non-mutant patients, *TP53*-mutant patients have a high expression of snoRNA family genes, and their DEGs are significantly enriched in several biological processes and pathways related to the regulation of rDNA and rRNA. SnoRNAs are a family of conserved RNAs, concentrated in Cajal bodies or nucleoli where they either function in the modification of rRNAs or participate in the processing of rRNAs during ribosomal subunit maturation (34). Many studies have shown that snoRNA is abnormally regulated in tumors (35–47), and snoRNA or snoRNA host genes can affect the proliferation, apoptosis, invasion and migration of cancer cells (37–47). Okugawa et al. and Mei et al. found snoRA42 enhance the proliferation, migration, invasion in colorectal cancer (CRC) and Lung cancer (41, 42). Fang et al. found snoRD126 activate the PI3k-AKT pathway to facilitate hepatocellular carcinoma (HCC) and CRC cell growth (43). Cui et al. found snoRA23 promote growth and metastasis by regulates expression of SYNE2 in pancreatic ductal adenocarcinoma (PDAC) (38). Valleron et al. found snoRD112-114 affects Rb/p16 cell cycle regulation to promote cell growth in acute promyelocytic leukemia (APL) (44). Siprashvili et al. found snoRD50A and snoRD50B activate the K-Ras/B-Raf-MEK-ERK pathway to facilitate the proliferation of tumor cells (45). Wu et al. found snoRNA Sf-15 can participate in apoptosis through regulating the expression of Ca2 + -induced cell death pathway gene Cn in Sf9 cells (46). Xia et al. found SNORD44 activate the caspase-dependent apoptosis pathway to facilitate the apoptosis in glioma cells (47). However, these studies are focused on the function of a single snoRNA. In this study, we found that snoRNA family genes are dysregulated expressed in clusters, rather than the disorder of a single snoRNA gene. Therefore, we speculate that snoRNA-mediated rRNA maturation, which is the unified function of snoRNA, might be a cause of cancer progression in patients with *TP53* mutations. Our prognostic analysis showed that upregulated snoRNA was significantly associated with poor prognosis in patients with PDAC. Experiments *in vitro* and *in vivo* have shown that the proportion of mature 18S rRNA and 28S rRNA is significantly reduced in p53 overexpressed PANC-1 and Patu-8988 pancreatic cancer cell lines, and knockdown of p53 in KP4 and PANC-0327 pancreatic cancer cell lines promoted the maturation of 18S rRNA and 28S rRNA. These results indicate that snoRNA-mediated rRNA maturation may be a possible mechanism for the progression of cancer in PDAC patients with *TP53* mutations, but we believe that snoRNA-mediated rRNA maturation is not simply a surrogate for proliferation rate, other targets and pathways affecting the proliferation of *TP53* mutant pancreatic cancer cells need further exploration, which is. a direction worthy of in-depth study in the future.

STRAP, a protein containing WD40 (48), is thought to play an important role in regulating eukaryotic cell growth and development by inhibiting transforming growth factor-beta (TGF-β) and various other signaling pathways (49–51). Recent studies have shown that overexpression and misregulation of STRAP are associated with the development of multiple cancers (52–54) and thus it could be considered a new therapeutic target for cancer. Our prognostic analysis showed that the expression of STRAP was significantly associated with the prognosis of PDAC patients. Krastev et al. (33) found that *TP53* can regulate immature snoRNPs into the Cajal body by regulating the level of NOLC1, and then immature snoRNPs interact with COIL and SMN to assemble mature snoRNPs. STRAP plays an important role in regulating the cellular localization of SMN complex, which is necessary for the SMN complex to enter Cajal body (55, 56). As a downstream concomitant factor affecting snoRNP assembly by *TP53*, the expression of STRAP is currently known to be independent of p53 expression. Our study also found that there was no significant difference in STRAP expression between PDAC patients with *TP53* mutation and patients with no *TP53* mutation. Krastev et al. (33) also showed that knocking down STRAP had no effect on the growth of *TP53* wild-type colon cancer cells, whereas expression of STRAP was required for efficient growth of *TP53* knockout colon cancer cells. This shows that STRAP as a target for adjuvant therapy may provide a huge advantage in terms of mitigating toxic and side effects on *TP53* non-mutated normal cells. Based on this, we successfully constructed STRAP-interfering pancreatic cancer cell lines with STRAP shRNA lentivirus, and verified their effects *in vitro* and *in vivo*. We found that knocking down STRAP could effectively inhibit rRNA maturation *in vitro* and *in vivo* and block the progression of pancreatic cancer cell lines with *TP53* mutations or p53 knockdown, while there was no significant effect on the pancreatic cancer cell lines with high p53 expression. Our study is the first to explore the effectiveness of STRAP in pancreatic cancer, providing a new target for the treatment of patients with poor prognosis in PDAC mainly caused by *TP53* mutation.

Taken together, our study identified the key contribution factor *TP53* that influenced the prognosis of PDAC based on a large sample analysis of public databases. In addition, we found a possible mechanism for disease progression in *TP53* mutant PDAC patients, and uncovered a new effective potential therapeutic target that can interfere with this pathway (Supplementary Figure 5). Our research provides reliable theoretical basis for precise classification and clinical adjuvant treatment of pancreatic cancer patients.

## Data Availability Statement

The raw data supporting the conclusions of this article will be made available by the authors, without undue reservation.

## Ethics Statement

The animal study was reviewed and approved by Institutional Animal Care and Use Committee of Wenzhou Medical University.

## Author Contributions

SH, XC, and QZ contributed to conception and design of the study. SH, XC, and XX organized the database. SH, XC, XX, CZ, WH, YZ, PA, HY, KS, and BC performed the experiments and the statistical analysis. SH, XC, PA, and QZ wrote the first draft of the manuscript. XX, CZ, WH, YZ, HY, KS, and BC wrote sections of the manuscript. All authors contributed to manuscript revision, read, and approved the submitted version.

## Conflict of Interest

The authors declare that the research was conducted in the absence of any commercial or financial relationships that could be construed as a potential conflict of interest.

## Footnotes

1 http://cancergenome.nih.gov/

2 https://dcc.icgc.org/

3 https://portals.broadinstitute.org/ccle
