# Supplementary material for: STRAP as a New Therapeutic Target for Poor Prognosis of Pancreatic Ductal Adenocarcinoma Patients Mainly Caused by TP53 Mutation
Source: Front Oncol. 2020 Sep 29;10:594224. doi: 10.3389/fonc.2020.594224 (PMC7550692; doi:10.3389/fonc.2020.594224)
Supplement: Supplementary file 1 [file Data_Sheet_1.pdf]

Supplementary Material

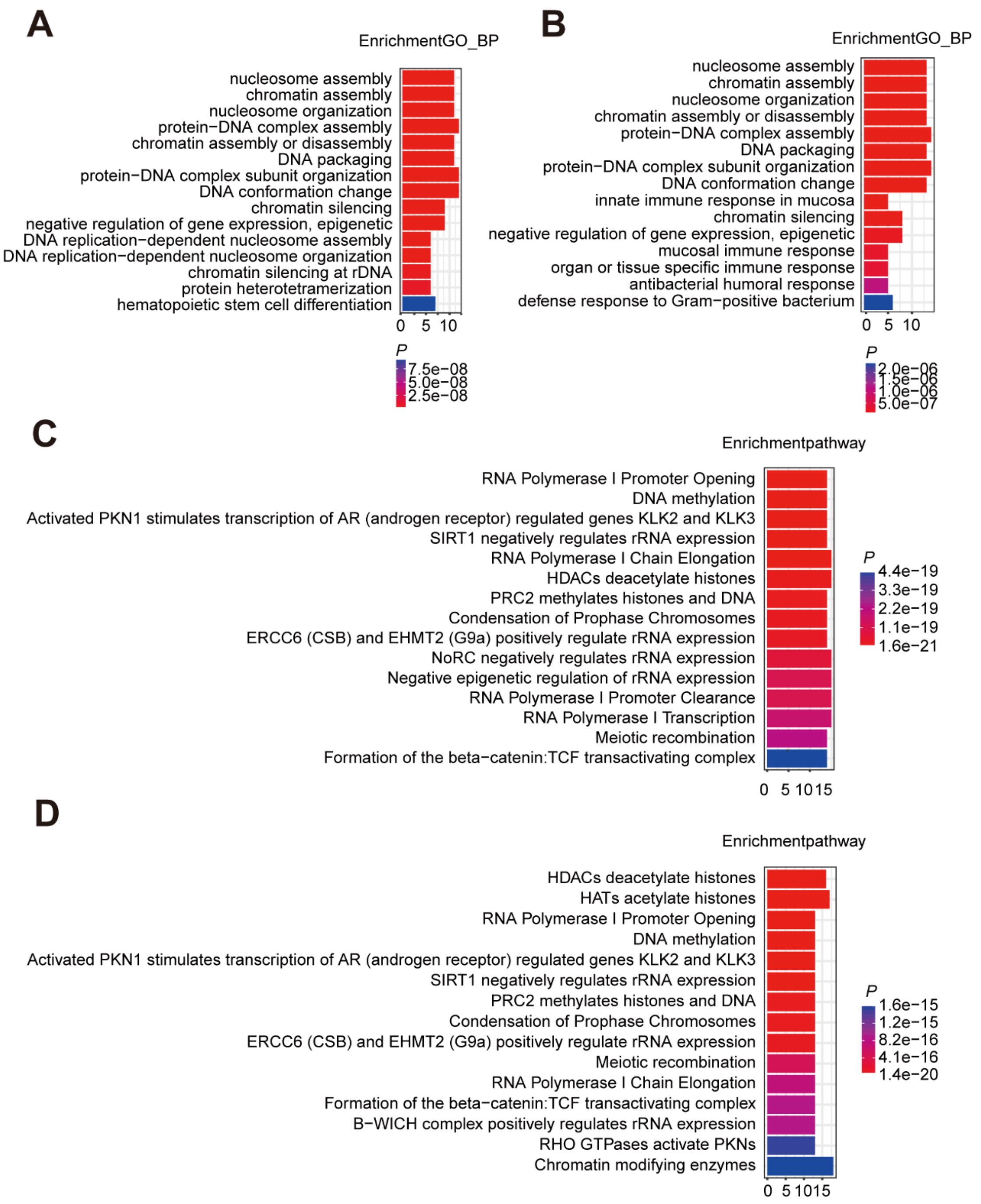

Supplementary Figure 1. Functional enrichment analysis of the differentially expressed genes (DEGs). (A) Biological process analysis of DEGs by Student's t-test. (B) Biological process analysis of DEGs by RankCompV2. (C) ReactomePA analysis of DEGs by Student's t-test. (D) ReactomePA analysis of DEGs by RankCompV2.

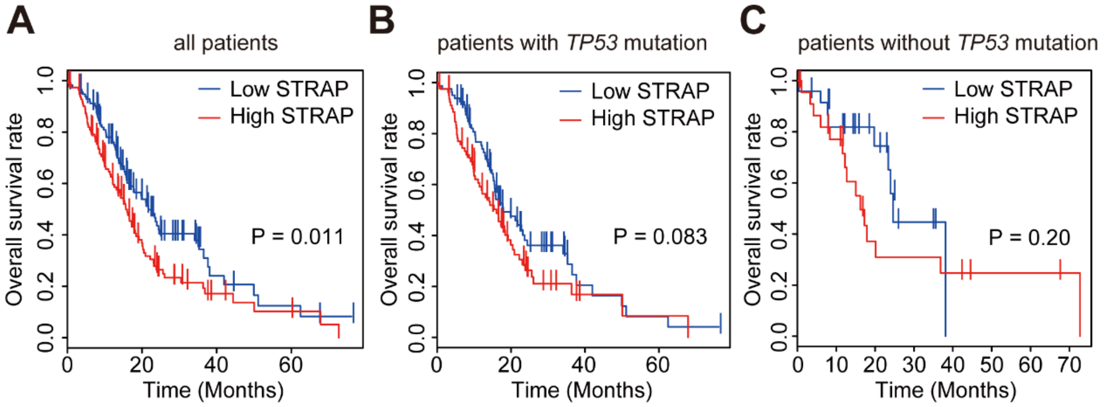

Supplementary Figure 2. Expression levels of STRAP were inversely correlated with the prognosis of PDAC patients. Kaplan–Meier survival curves for PDAC patients according to the STRAP expression in tumor tissues based on (A) all patients, (B) patients with *TP53* mutation and (C) patients without *TP53* mutation. Significance was calculated using the log-rank test.

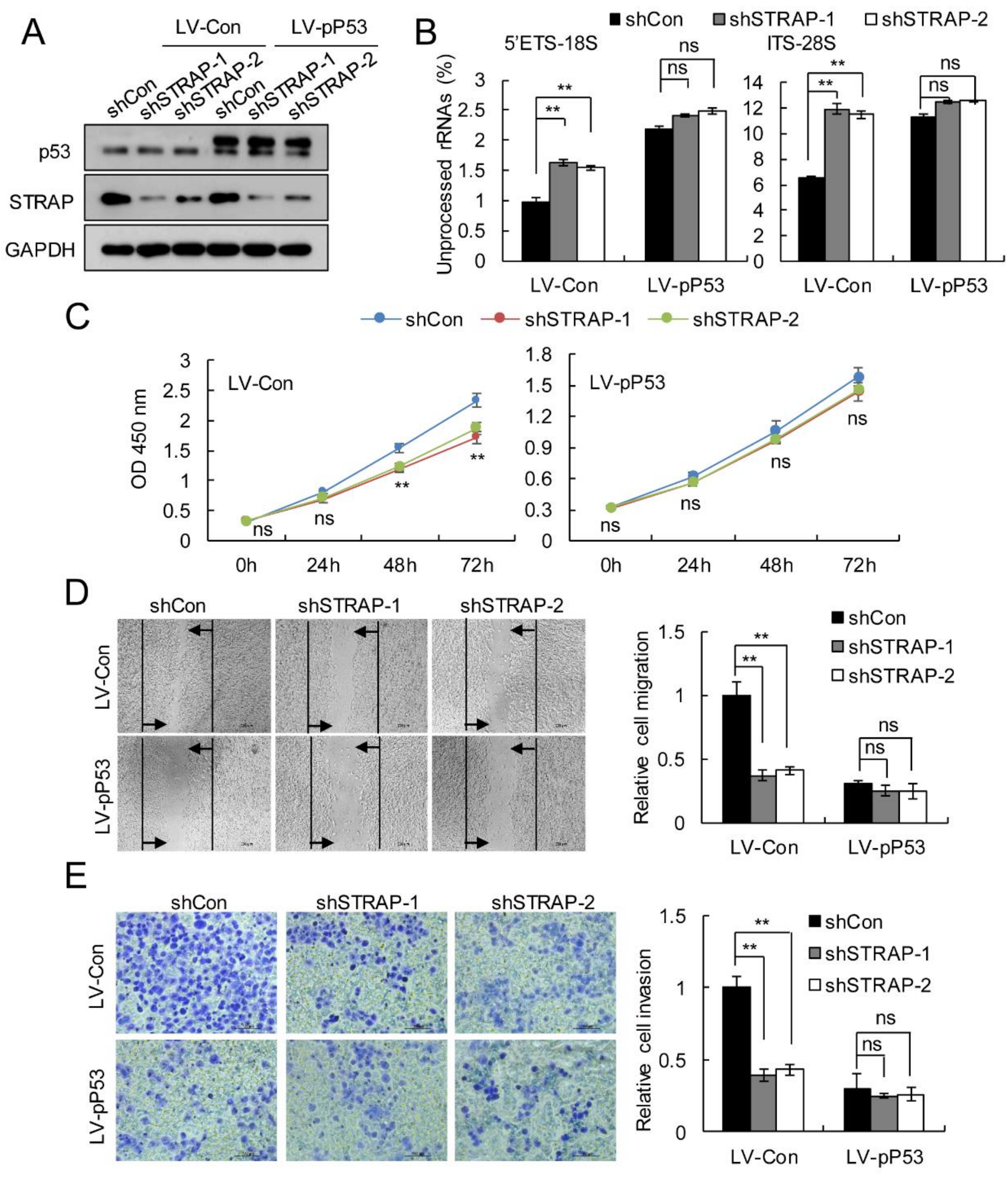

Supplementary Figure 3. STRAP knockdown suppressed progression of *TP53*-mutant PANC-1 cells by inhibiting snoRNA-mediated rRNA maturation. PANC-1 cells with p53 overexpression or control vector were infected with control or STRAP-knockdown lentiviruses. (A) p53 and STRAP expression were analyzed by western blotting. (B) rRNA processing by detecting 5'ETS-18S and ITS–28S using quantitative reverse transcriptase PCR assays. The data are shown as the mean ± SEM of 3 independent experiments. (C) The cell proliferation assay was performed at the indicated time points. (D) Representative micrographs of cell migration assays at 48h (left) and quantification results (right). (E) Representative micrographs of cell invasion assays (left) and quantification results (right). Data in C-E are shown as the mean ± SEM of 3 independent experiments. $^{**}P < 0.01$.

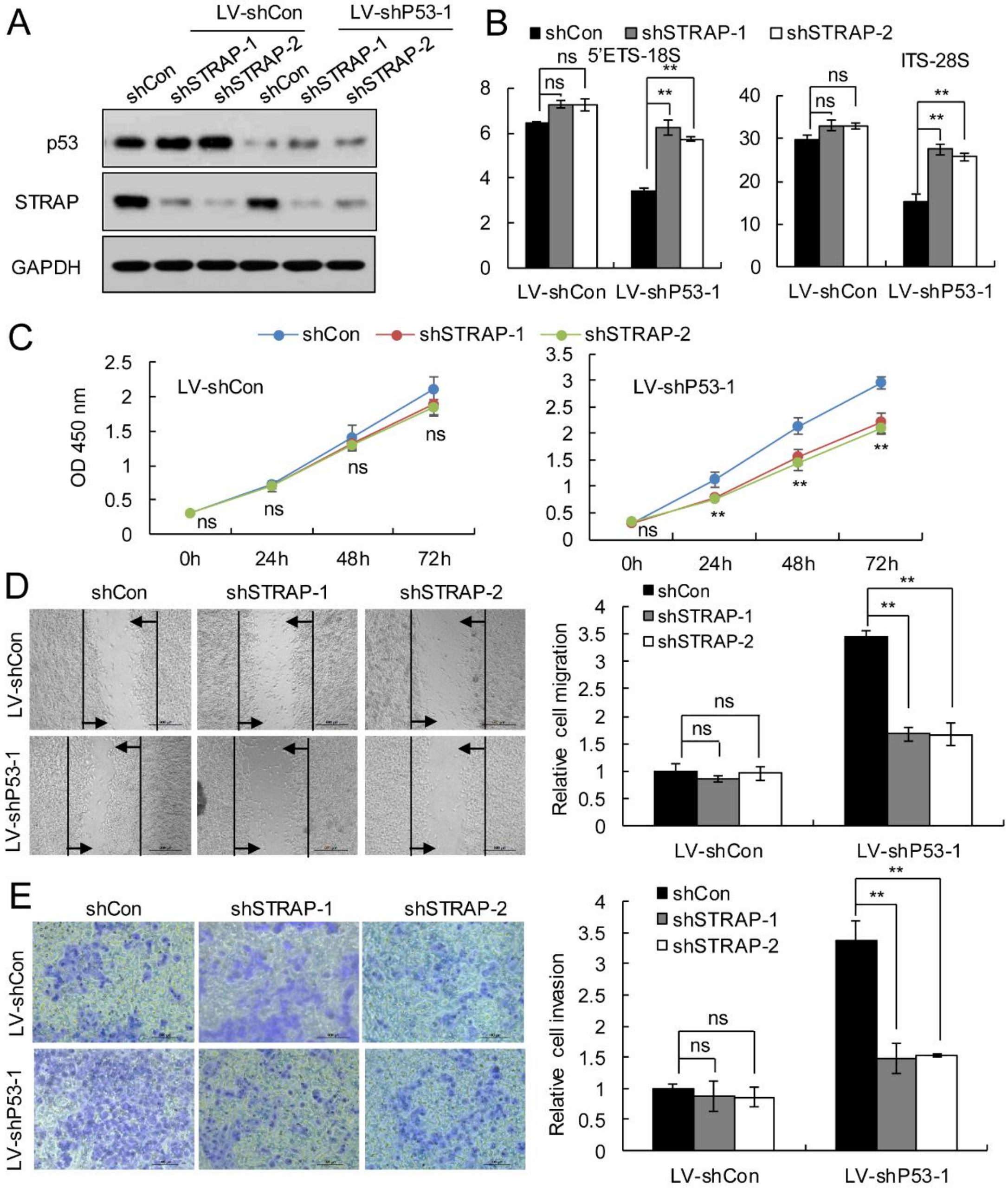

Supplementary Figure 4. STRAP knockdown suppressed progression of p53-knockdown PANC-0327 cells by inhibiting snoRNA-mediated rRNA maturation. PANC-0327 cells with p53 knockdown or control vector were infected with control or STRAP-knockdown lentiviruses. (A) p53 and STRAP expression were analyzed by western blotting. (B) rRNA processing by detecting 5'ETS-18S and ITS–28S using quantitative reverse transcriptase PCR assays. The data are shown as the mean ± SEM of 3 independent experiments. (C) The cell proliferation assay was performed at the indicated time points. (D) Representative micrographs of cell migration assays at 48h (left) and quantification results (right). (E) Representative micrographs of cell invasion assays (left) and quantification results (right). Data in C-E are shown as the mean ± SEM of 3 independent experiments. $^{**}P < 0.01$.

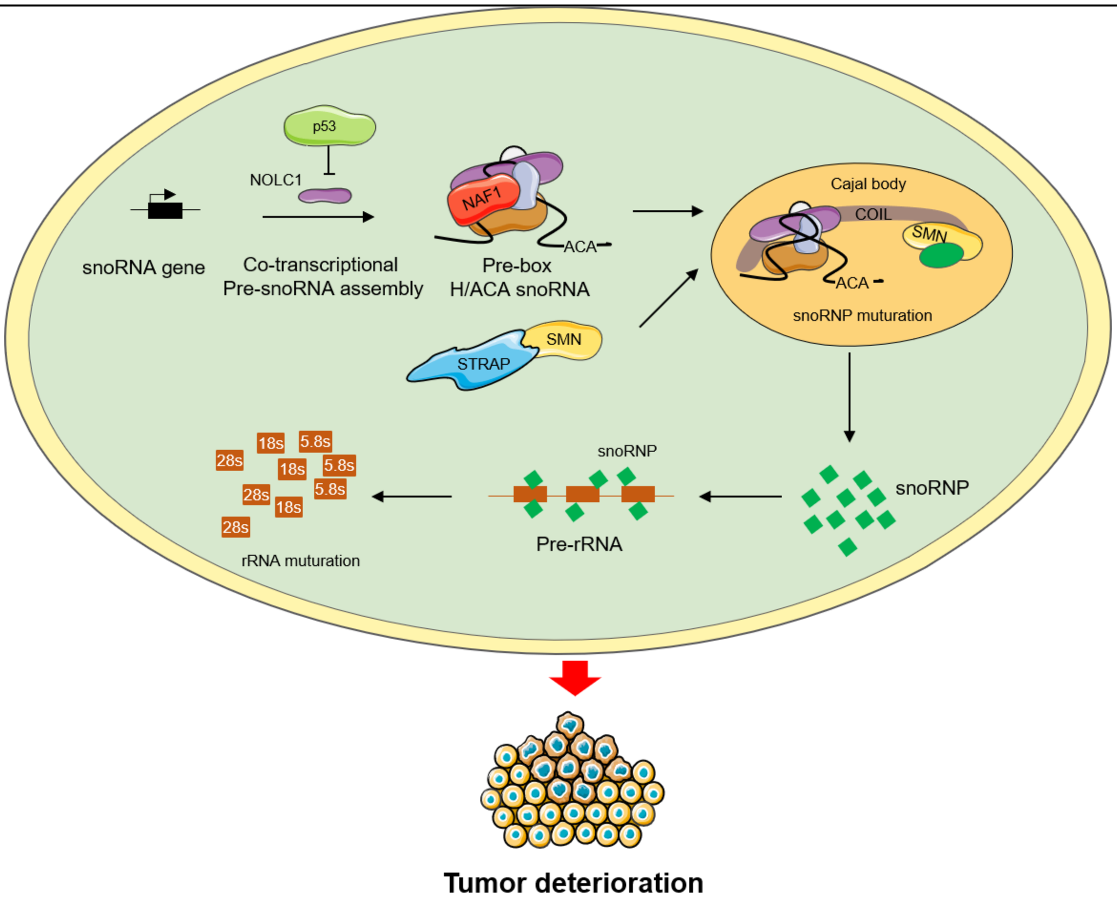

Supplementary Figure 5. Mechanism model of TP53 affecting PDAC progression.

Supplementary Tables

Supplementary Table 1. Sequences of primers for qPCR.

Primers[a]	Sequences (5′ → 3′)	Usage (Target loci/genes[b])
H-GAPDH-F	ATGACATCAAGAAGGTGGTG	GAPDH
H-GAPDH-R	CATACCAGGAAATGAGCTTG	
18S-1	GCGGGACACTCAGCTAAGAGC	Quantification for both processed and unprocessed 18S rRNA
18S-2	GGCCCTGTAATTGGAATGAG	
18S-3	GCGCCCGTCGGCATGTATTAGCTC	Quantification for unprocessed 18S rRNA using an sense primer
18S-4	CTCGCCGCGCTCTACCTTACCTACCTGG	
18S-5	GGCAGGATCAACCAGGTAGGTAAGG	Quantification for unprocessed 18S rRNAusing an anti-sense primer
18S-6	GGGGGGCGGGTGGTTGGGGCGTCC	
28S-a	CTTACGGTACTTGTTGACTATCGGTCTCG	Quantification for both processed and unprocessed 28S rRNA
28S-b	CCAAGTCCTTCTGATCGAGGCCC	
28S-c	CGCTGGGCTCTTCCCTGTTCACTCG	Quantification for unprocessed 28S rRNA using a specific sense primer
28S-d	CCCGTCCCCCTCCGAGACGCGACC	
28S-e	CGTCTGATCTGAGGTCGCGTCTCGG	Quantification for unprocessed 28S rRNA using an anti-sense primer
28S-f	CTCTCTCCCGTCGCCTCTCCCC	

[a] F, forward strand; R, reverse strand.

Supplementary Table 2. Information of donors in the data sets.

Data sets	Country	Donors	SSM	Effective donor	RNA-seq
PACA-AU	Australia	461	391	386	91
PACA-CA	Canada	308	260	247	46
PAAD-AU (TCGA)	United States	154	133	133	142
Total	-	923	784	762	279

SSM: Simple Somatic Mutation

Supplementary Table 3. Multivariate analysis of factors affecting patients' overall survival in PDAC (n = 762).

Factors	Patients	Median Survival (Months)	*P*
Platform	762	19.9	0.58
TCGA	133	19.9	
PACA-AU	386	18.7	
PACA-CA	243	20.9	
Gender	762	19.9	0.23
Male	417	19.2	
Female	345	20.3	
Age	758	19.9	0.076
>65 year	407	18.4	
<=65 year	351	21.6	
Mutation			0.0028
0	38	29.8	
1	153	24.6	
2	343	19.9	
3	198	17.2	
4	30	15.2	

Supplementary Table 4. Mutation frequency of four driver genes in patients with PDAC in this study.

Mutation	Total patients	KRAS(%)	TP53(%)	SMAD4(%)	CDKN2A(%)
/	784	709(90.43)	542(69.13)	182(23.21)	162(20.66)
1 gene	159(20.28)	131(82.39)	22(13.84)	4(2.52)	2(1.26)
2 genes	353(45.03)	347(98.30)	293(83.00)	46(13.03)	20(5.67)
3 genes	202(25.77)	200(99.01)	196(97.03)	101(50.00)	109(53.96)
>=1 genes	745(95.03)	709(95.17)	542(72.75)	182(24.43)	162(21.74)
>=2 genes	586(74.74)	578(98.63)	520(88.74)	178(30.38)	160(27.30)
>=3 genes	233(29.72)	231(99.14)	227(97.42)	132(56.65)	140(60.09)

Supplementary Table 5. Mutation background in the four driver genes of pancreatic cancer cell lines in CCLE.

Cell Type	Hugo Symbol	Entrez Gene Id	Ncbi Build	Chr	Start Position	End Position	Reference Allele	Tumor Seq Allele	Protein Change
PANC-1	KRAS	3845	37	12	25398284	25398284	C	T	p.G12D
	TP53	7157	37	17	7577120	7577120	C	T	p.R273H
Patu-8988	KRAS	3845	37	12	25398284	25398284	C	A	p.G12V
	TP53	7157	37	17	7577094	7577094	G	A	p.R282W
KP4	KRAS	3845	37	12	25398284	25398284	C	T	p.G12D
PANC-0327	KRAS	3845	37	12	25398284	25398284	C	A	p.G12V

Supplementary Table 6. Differentially expressed genes in patients with *TP53* mutation compared to non-mutated patients.

Gene symbol	Enterz id	*P*	OR	Description	Category	Gene symbol	Enterz id	Direction	Description	Category
Student's two-tailed t-test						RankCompV2				
ADAM6	8755	2.13E-02	-2.41	ADAM Metallopeptidase Domain 6 (Pseudogene)	Pseudogene	ADH1C	126	down	Alcohol Dehydrogenase 1C (Class I), Gamma Polypeptide	Protein Coding
ADAMTSL3	57188	7.19E-03	2.41	ADAMTS Like 3	Protein Coding	ADRA2A	150	down	Adrenoceptor Alpha 2A	Protein Coding
ADH1B	125	2.88E-02	-2.20	Alcohol Dehydrogenase 1B (Class I), Beta Polypeptide	Protein Coding	AQP1	358	down	Aquaporin 1 (Colton Blood Group)	Protein Coding
AGAP11	119385	1.37E-02	2.70	ArfGAP With GTPase Domain, Ankyrin Repeat And PH Domain 11	Protein Coding	C7orf71	285941	down	Chromosome 7 Open Reading Frame 71	Protein Coding

ANKRD36B	57730	1.40E-03	2.43	Ankyrin Repeat Domain 36B	Protein Coding	CAMTA2	23125	down	Calmodulin Binding Transcription Activator 2	Protein Coding
ASTN2	23245	3.69E-02	3.07	Astrotactin 2	Protein Coding	CHKB-CPT1B	386593	down	CHKB-CPT1B Readthrough (NMD Candidate)	RNA Gene
ATP9B	374868	2.60E-03	2.87	ATPase Phospholipid Transporting 9B (Putative)	Protein Coding	CLDN2	9075	down	Claudin 2	Protein Coding
C12orf50	160419	1.04E-02	4.06	Chromosome 12 Open Reading Frame 50	Protein Coding	CLDN5	7122	down	Claudin 5	Protein Coding
C16orf74	404550	3.00E-02	2.04	Chromosome 16 Open Reading Frame 74	Protein Coding	CRISP3	10321	down	Cysteine Rich Secretory Protein 3	Protein Coding
C4orf36	132989	8.61E-04	2.63	Chromosome 4 Open Reading Frame 36	Protein Coding	CTRB2	440387	down	Chymotrypsinogen B2	Protein Coding

C7	730	2.11E-02	-2.14	Complement C7	Protein Coding	DCAF8	50717	down	DDB1 And CUL4 Associated Factor 8	Protein Coding
CBWD5	220869	7.38E-04	2.23	COBW Domain Containing 5	Protein Coding	FGFR4	2264	down	Fibroblast Growth Factor Receptor 4	Protein Coding
CD3E	916	4.68E-02	-2.04	CD3e Molecule	Protein Coding	HPYR1	93668	down	Helicobacter Pylori Responsive 1	RNA Gene
CGB5	93659	4.87E-02	3.16	Chorionic Gonadotropin Subunit Beta 5	Protein Coding	HYAL1	3373	down	Hyaluronoglucosami nidase 1	Protein Coding
CHRDL1	91851	1.59E-02	-2.42	Chordin Like 1	Protein Coding	KDM6B	23135	down	Lysine Demethylase 6B	Protein Coding
CLDN2	9075	1.10E-03	-2.28	Claudin 2	Protein Coding	LIPI	149998	down	Lipase I	Protein Coding
COX6B2	125965	7.00E-05	2.07	Cytochrome C Oxidase Subunit 6B2	Protein Coding	LYG2	254773	down	Lysozyme G2	Protein Coding
CYSLTR1	10800	2.68E-03	3.77	Cysteinyl Leukotriene Receptor 1	Protein Coding	MUC6	4588	down	Mucin 6, Oligomeric Mucus/Gel-Forming	Protein Coding

DARC	2532	9.70E-03	-2.03	Atypical Chemokine Receptor 1 (Duffy Blood Group)	Protein Coding	NPC1L1	29881	down	NPC1 Like Intracellular Cholesterol Transporter 1	Protein Coding
DNAH2	146754	4.88E-03	-2.35	Dynein Axonemal Heavy Chain 2	Protein Coding	NPHS2	7827	down	NPHS2, Podocin	Protein Coding
DPT	1805	1.11E-02	-2.02	Dermatoponti n	Protein Coding	OR2A25	392138	down	Olfactory Receptor Family 2 Subfamily A Member 25	Protein Coding
FABP5	2171	7.50E-03	2.50	Fatty Acid Binding Protein 5	Protein Coding	OR5K2	402135	down	Olfactory Receptor Family 5 Subfamily K Member 2	Protein Coding
FST	10468	4.70E-02	2.30	Follistatin	Protein Coding	SMOC2	64094	down	SPARC Related Modular Calcium Binding 2	Protein Coding

GTF2H2	2966	1.24E-04	2.02	General Transcription Factor IIH Subunit 2	Protein Coding	SNORA8	654320	down	Small Nucleolar RNA, H/ACA Box 8	RNA Gene
GUSBP3	653188	2.06E-03	2.54	Glucuronidase , Beta Pseudogene 3	Pseudogene	SOX1	6656	down	SRY-Box 1	Protein Coding
HES4	57801	5.59E-03	2.12	Hes Family BHLH Transcription Factor 4	Protein Coding	TCL1B	9623	down	T Cell Leukemia/Lymphom a 1B	Protein Coding
HIST1H2 AD	3013	4.53E-03	2.23	Histone Cluster 1 H2A Family Member D	Protein Coding	TMBIM4	51643	down	Transmembrane BAX Inhibitor Motif Containing 4	Protein Coding
HIST1H2 AE	3012	4.16E-03	3.12	Histone Cluster 1 H2A Family Member E	Protein Coding	TNXB	7148	down	Tenascin XB	Protein Coding

HIST1H2 AJ	8331	2.06E-02	2.24	Histone Cluster 1 H2A Family Member J	Protein Coding	TRPC7	57113	down	Transient Receptor Potential Cation Channel Subfamily C Member 7	Protein Coding
HIST1H2 AK	8330	2.57E-02	2.07	Histone Cluster 1 H2A Family Member K	Protein Coding	TYR	7299	down	Tyrosinase	Protein Coding
HIST1H2 BF	8343	1.76E-02	2.70	Histone Cluster 1 H2B Family Member F	Protein Coding	ADK	132	up	Adenosine Kinase	Protein Coding
HIST1H2 BG	8339	5.17E-04	3.34	Histone Cluster 1 H2B Family Member G	Protein Coding	ARNTL2	56938	up	Aryl Hydrocarbon Receptor Nuclear Translocator Like 2	Protein Coding
HIST1H2 BH	8345	5.26E-03	4.67	Histone Cluster 1 H2B Family Member H	Protein Coding	ATP5I	521	up	ATP Synthase Membrane Subunit E	Protein Coding

HIST1H2BI	8346	1.45E-02	3.19	Histone Cluster 1 H2B Family Member I	Protein Coding	BCYRN1	618	up	Brain Cytoplasmic RNA 1	RNA Gene
HIST1H2BM	8342	1.71E-02	2.93	Histone Cluster 1 H2B Family Member M	Protein Coding	C12orf50	160419	up	Chromosome 12 Open Reading Frame 50	Protein Coding
HIST1H3D	8351	2.75E-02	2.05	Histone Cluster 1 H3 Family Member D	Protein Coding	C15orf48	84419	up	Chromosome 15 Open Reading Frame 48	Protein Coding
HIST1H3F	8968	5.66E-03	5.55	Histone Cluster 1 H3 Family Member F	Protein Coding	C8orf59	401466	up	Chromosome 8 Open Reading Frame 59	Protein Coding
HIST1H3J	8356	2.60E-02	2.15	Histone Cluster 1 H3 Family Member J	Protein Coding	CARD18	59082	up	Caspase Recruitment Domain Family Member 18	Protein Coding

HIST1H4E	8367	1.41E-02	2.27	Histone Cluster 1 H4 Family Member E	Protein Coding	CBWD6	644019	up	COBW Domain Containing 6	Protein Coding
HIST1H4J	8363	2.57E-02	2.13	Histone Cluster 1 H4 Family Member J	Protein Coding	CDHR4	389118	up	Cadherin Related Family Member 4	Protein Coding
HIST1H4K	8362	9.20E-04	3.40	Histone Cluster 1 H4 Family Member K	Protein Coding	DNAJC25-GNG10	552891	up	DNAJC25-GNG10 Readthrough	Protein Coding
HRASLS2	54979	2.61E-02	2.79	HRAS Like Suppressor 2	Protein Coding	EIF3C	8663	up	Eukaryotic Translation Initiation Factor 3 Subunit C	Protein Coding
HSPB6	126393	4.50E-02	-2.46	Heat Shock Protein Family B (Small) Member 6	Protein Coding	ENY2	56943	up	ENY2, Transcription And Export Complex 2 Subunit	Protein Coding

IGF1	3479	3.82E-02	-2.06	Insulin Like Growth Factor 1	Protein Coding	FABP6	2172	up	Fatty Acid Binding Protein 6 Protein Coding	Protein Coding
KIAA1328	57536	1.18E-03	3.88	KIAA1328	Protein Coding	FAM9B	171483	up	Family With Sequence Similarity 9 Member B	Protein Coding
KRT13	3860	4.61E-02	2.86	Keratin 13	Protein Coding	GNGT1	2792	up	G Protein Subunit Gamma Transducin 1	Protein Coding
KRT6A	3853	4.56E-03	3.14	Keratin 6A	Protein Coding	HIST1H1C	3006	up	Histone Cluster 1 H1 Family Member C	Protein Coding
LYPD3	27076	2.46E-03	2.06	LY6/PLAUR Domain Containing 3	Protein Coding	HIST1H2AB	8335	up	Histone Cluster 1 H2A Family Member B	Protein Coding

MALAT1	378938	1.29E-04	2.75	Metastasis Associated Lung Adenocarcinoma Transcript 1	RNA Gene	HIST1H2AC	8334	up	Histone Cluster 1 H2A Family Member C	Protein Coding
MIPOL1	145282	9.88E-03	2.53	Mirror-Image Polydactyly 1	Protein Coding	HIST1H2AM	8336	up	Histone Cluster 1 H2A Family Member M	Protein Coding
MMRN1	22915	1.08E-02	-2.02	Multimerin 1	Protein Coding	HIST1H2BB	3018	up	Histone Cluster 1 H2B Family Member B	Protein Coding
NAV3	89795	1.16E-02	2.38	Neuron Navigator 3	Protein Coding	HIST1H2BC	8347	up	Histone Cluster 1 H2B Family Member C	Protein Coding
NEDD4	4734	8.46E-04	3.74	Neural Precursor Cell Expressed, Developmentally Down-Regulat	Protein Coding	HIST1H2BI	8346	up	Histone Cluster 1 H2B Family Member I	Protein Coding

				ed 4, E3 Ubiquitin Protein Ligase						
P2RY8	286530	4.01E-02	-2.01	P2Y Receptor Family Member 8	Protein Coding	HIST1H2BJ	8970	up	Histone Cluster 1 H2B Family Member J	Protein Coding
PARP15	165631	9.90E-03	2.45	Poly(ADP-Ribose) Polymerase Family Member 15	Protein Coding	HIST1H2BM	8342	up	Histone Cluster 1 H2B Family Member M	Protein Coding
PI3	5266	5.20E-03	2.16	Peptidase Inhibitor 3	Protein Coding	HIST1H2BO	8348	up	Histone Cluster 1 H2B Family Member C	Protein Coding
PIGL	9487	8.00E-04	2.49	Phosphatidylinositol Glycan Anchor Biosynthesis Class L	Protein Coding	HIST1H3B	8358	up	Histone Cluster 1 H3 Family Member B	Protein Coding

PLIN4	729359	2.66E-02	-3.21	Perilipin 4	Protein Coding	HIST1H3H	8357	up	Histone Cluster 1 H3 Family Member H	Protein Coding
POP4	10775	1.88E-02	2.11	POP4 Homolog, Ribonuclease P/MRP Subunit	Protein Coding	HIST1H4H	8365	up	Histone Cluster 1 H4 Family Member H	Protein Coding
PPP1R16 B	26051	2.81E-02	-2.12	Protein Phosphatase 1 Regulatory Subunit 16B	Protein Coding	HIST2H2AB	317772	up	Histone Cluster 2 H2A Family Member B	Protein Coding
PSCA	8000	9.63E-04	2.19	Prostate Stem Cell Antigen	Protein Coding	HIST2H2BE	8349	up	Histone Cluster 2 H2B Family Member E	Protein Coding
PVT1	5820	2.05E-05	2.34	Pvt1 Oncogene	RNA Gene	HIST2H2BF	440689	up	Histone Cluster 2 H2B Family Member F	Protein Coding

RIC3	79608	1.37E-02	2.51	RIC3 Acetylcholine Receptor Chaperone	Protein Coding	HIST2H4A	8370	up	Histone Cluster 2 H4 Family Member A	Protein Coding
ROMO1	140823	2.33E-02	2.23	Reactive Oxygen Species Modulator 1	Protein Coding	HTN3	3347	up	Histatin 3	Protein Coding
RPS28	6234	9.49E-03	2.49	Ribosomal Protein S28	Protein Coding	IFI44	10561	up	Interferon Induced Protein 44	Protein Coding
SCARNA 5	677775	6.77E-03	2.08	mall Cajal Body-Specific RNA 5	RNA Gene	IGF2BP3	10643	up	Insulin Like Growth Factor 2 MRNA Binding Protein 3	Protein Coding
SCARNA 9	619383	2.49E-03	2.16	Small Cajal Body-Specific RNA 9	RNA Gene	KRTAP4-1	85285	up	Keratin Associated Protein 4-1	Protein Coding

SFRP1	6422	2.98E-02	-2.04	Secreted Frizzled Related Protein 1	Protein Coding	MBOAT2	129642	up	Membrane Bound O-Acyltransferase Domain Containing 2	Protein Coding
SHISA9	729993	3.84E-03	3.37	Shisa Family Member 9	Protein Coding	MRGPRX3	117195	up	MAS Related GPR Family Member X3	Protein Coding
SNCG	6623	1.54E-02	2.72	Synuclein Gamma	Protein Coding	NKX2-1	7080	up	NK2 Homeobox 1	Protein Coding
SNORA12	677800	1.87E-04	3.48	Small Nucleolar RNA, H/ACA Box 12	RNA Gene	NSMCE2	286053	up	NSE2 (MMS21) Homolog, SMC5-SMC6 Complex SUMO Ligase	Protein Coding

SNORA37	677819	1.89E-02	3.59	Small Nucleolar RNA, H/ACA Box 37	RNA Gene	OR1J4	26219	up	Olfactory Receptor Family 1 Subfamily J Member 4	Protein Coding
SNORA38B	100124536	8.04E-03	2.84	Small Nucleolar RNA, H/ACA Box 38B	RNA Gene	PDCD5	9141	up	Programmed Cell Death 5	Protein Coding
SNORA47	677828	4.00E-03	4.27	Small Nucleolar RNA, H/ACA Box 47	RNA Gene	PIWIL3	440822	up	Piwi Like RNA-Mediated Gene Silencing 3	Protein Coding
SNORA49	677829	4.19E-02	2.77	Small Nucleolar RNA, H/ACA Box 49	RNA Gene	PSCA	8000	up	Prostate Stem Cell Antigen	Protein Coding
SNORA5A	654319	4.26E-02	5.71	Small Nucleolar RNA, H/ACA Box 5A	RNA Gene	PSMC1	5700	up	Proteasome 26S Subunit, ATPase 1	Protein Coding
SNORA74A	26821	7.36E-03	2.34	Small Nucleolar RNA, H/ACA Box 74A	RNA Gene	RANBP1	5902	up	RAN Binding Protein 1	Protein Coding

SNORA74B	677841	1.17E-03	2.81	Small Nucleolar RNA, H/ACA Box 74B	RNA Gene	RARRES3	5920	up	Retinoic Acid Receptor Responder 3	Protein Coding
SNORA79	677845	7.07E-03	3.13	Small Nucleolar RNA, H/ACA Box 79	RNA Gene	RGPD8	727851	up	RANBP2-Like And GRIP Domain Containing 8	Protein Coding
SNORA80	677846	4.41E-02	2.59	Small Nucleolar RNA, H/ACA Box 80D	RNA Gene	RPL39	6170	up	Ribosomal Protein L39	Protein Coding
SNORD15B	114599	2.35E-02	4.55	Small Nucleolar RNA, C/D Box 15B	RNA Gene	RPS28	6234	up	Ribosomal Protein S28	Protein Coding
SNORD17	692086	2.37E-03	2.21	Small Nucleolar RNA, C/D Box 17	RNA Gene	SCARNA1	677774	up	Small Cajal Body-Specific RNA 1	RNA Gene
SPC25	57405	2.50E-04	2.06	SPC25, NDC80 Kinetochore Complex Component	Protein Coding	SCARNA23	677773	up	Small Cajal Body-Specific RNA 23	RNA Gene

TBC1D8B	54885	4.05E-03	2.43	TBC1 Domain Family Member 8B	Protein Coding	SCARNA5	677775	up	Small Cajal Body-Specific RNA 5	RNA Gene
TMEM160	54958	7.12E-03	2.03	Transmembrane Protein 160	Protein Coding	SCEL	8796	up	Sciellin	Protein Coding
TMEM182	130827	3.81E-03	3.09	Transmembrane Protein 182	Protein Coding	SNORA38B	100124536	up	Small Nucleolar RNA, H/ACA Box 38B	RNA Gene
TMEM212	389177	1.07E-02	4.38	Transmembrane Protein 212	Protein Coding	SNORA71D	677840	up	Small Nucleolar RNA, H/ACA Box 71D	RNA Gene
TMEM45A	55076	5.92E-03	2.52	Transmembrane Protein 45A	Protein Coding	SNORA79	677845	up	Small Nucleolar RNA, H/ACA Box 79	RNA Gene
TNXB	7148	2.72E-03	-2.04	Tenascin XB	Protein Coding	SPERT	220082	up	Spermatid Associated	Protein Coding
WNT11	7481	7.24E-03	2.05	Wnt Family Member 11	Protein Coding	SPINK9	643394	up	Spermatid Associated	Protein Coding

Supplementary Table 7. Functional enrichment analysis of differentially expressed genes identified by Student's t-test.

Category	ID	Description	*P*	p.adjust	qvalue	Count
BP	GO:0006334	nucleosome assembly	1.63E-12	2.11E-09	1.88E-09	11
BP	GO:0031497	chromatin assembly	6.25E-12	4.04E-09	3.60E-09	11
BP	GO:0034728	nucleosome organization	1.19E-11	5.12E-09	4.56E-09	11
BP	GO:0065004	protein-DNA complex assembly	2.05E-11	6.61E-09	5.90E-09	12
BP	GO:0006333	chromatin assembly or disassembly	2.90E-11	7.50E-09	6.69E-09	11
BP	GO:0006323	DNA packaging	7.70E-11	1.54E-08	1.37E-08	11
BP	GO:0071824	protein-DNA complex subunit organization	8.31E-11	1.54E-08	1.37E-08	12
BP	GO:0071103	DNA conformation change	1.67E-10	2.60E-08	2.32E-08	12
BP	GO:0006342	chromatin silencing	1.81E-10	2.60E-08	2.32E-08	9
BP	GO:0045814	negative regulation of gene expression, epigenetic	5.68E-10	7.34E-08	6.54E-08	9
BP	GO:0006335	DNA replication-dependent nucleosome assembly	8.97E-10	9.66E-08	8.61E-08	6
BP	GO:0034723	DNA replication-dependent nucleosome organization	8.97E-10	9.66E-08	8.61E-08	6
BP	GO:0000183	chromatin silencing at rDNA	2.69E-09	2.68E-07	2.39E-07	6
BP	GO:0051290	protein heterotetramerization	6.77E-09	6.26E-07	5.58E-07	6
BP	GO:0060218	hematopoietic stem cell differentiation	9.31E-08	8.02E-06	7.15E-06	7
BP	GO:0045652	regulation of megakaryocyte differentiation	1.93E-07	1.48E-05	1.32E-05	6
BP	GO:0002244	hematopoietic progenitor cell differentiation	1.95E-07	1.48E-05	1.32E-05	8
BP	GO:0045815	positive regulation of gene expression, epigenetic	2.63E-07	1.89E-05	1.68E-05	6
BP	GO:0030219	megakaryocyte differentiation	6.45E-07	4.39E-05	3.91E-05	6
BP	GO:1902036	regulation of hematopoietic stem cell differentiation	1.18E-06	7.62E-05	6.80E-05	6
BP	GO:0060964	regulation of gene silencing by miRNA	1.40E-06	8.60E-05	7.66E-05	6
BP	GO:0060147	regulation of posttranscriptional gene silencing	1.64E-06	9.24E-05	8.24E-05	6
BP	GO:0060966	regulation of gene silencing by RNA	1.64E-06	9.24E-05	8.24E-05	6
BP	GO:1901532	regulation of hematopoietic progenitor cell differentiation	2.03E-06	1.09E-04	9.75E-05	6

BP	GO:0051291	protein heterooligomerization	3.03E-06	1.57E-04	1.40E-04	6
BP	GO:0060968	regulation of gene silencing	4.80E-06	2.39E-04	2.13E-04	6
BP	GO:2000736	regulation of stem cell differentiation	7.33E-06	3.51E-04	3.13E-04	6
BP	GO:0051262	protein tetramerization	8.95E-06	4.13E-04	3.69E-04	6
BP	GO:0016458	gene silencing	1.14E-05	5.08E-04	4.53E-04	9
BP	GO:0045637	regulation of myeloid cell differentiation	1.53E-05	6.58E-04	5.87E-04	7
BP	GO:0032200	telomere organization	1.99E-05	8.31E-04	7.40E-04	6
BP	GO:0048863	stem cell differentiation	2.49E-05	1.00E-03	8.95E-04	7
BP	GO:0019730	antimicrobial humoral response	2.60E-05	1.02E-03	9.07E-04	5
BP	GO:0045653	negative regulation of megakaryocyte differentiation	2.78E-05	1.06E-03	9.41E-04	3
BP	GO:0040029	regulation of gene expression, epigenetic	2.96E-05	1.09E-03	9.75E-04	9
BP	GO:0061844	antimicrobial humoral immune response mediated by antimicrobial peptide	3.12E-05	1.12E-03	9.98E-04	4
BP	GO:0002227	innate immune response in mucosa	5.19E-05	1.81E-03	1.62E-03	3
BP	GO:0002385	mucosal immune response	1.63E-04	5.56E-03	4.95E-03	3
BP	GO:0002251	organ or tissue specific immune response	1.96E-04	6.50E-03	5.79E-03	3
BP	GO:0045638	negative regulation of myeloid cell differentiation	2.11E-04	6.83E-03	6.09E-03	4
BP	GO:0050830	defense response to Gram-positive bacterium	2.40E-04	7.58E-03	6.76E-03	4
BP	GO:0038111	interleukin-7-mediated signaling pathway	2.96E-04	9.11E-03	8.12E-03	3
BP	GO:0030099	myeloid cell differentiation	3.05E-04	9.16E-03	8.16E-03	7
BP	GO:0098760	response to interleukin-7	3.19E-04	9.17E-03	8.17E-03	3
BP	GO:0098761	cellular response to interleukin-7	3.19E-04	9.17E-03	8.17E-03	3
BP	GO:0034080	CENP-A containing nucleosome assembly	3.69E-04	1.02E-02	9.05E-03	3
BP	GO:0061641	CENP-A containing chromatin organization	3.69E-04	1.02E-02	9.05E-03	3
BP	GO:0019731	antibacterial humoral response	4.24E-04	1.12E-02	9.97E-03	3
BP	GO:1904837	beta-catenin-TCF complex assembly	4.24E-04	1.12E-02	9.97E-03	3

BP	GO:0031055	chromatin remodeling at centromere	4.83E-04	1.25E-02	1.11E-02	3
BP	GO:0035195	gene silencing by miRNA	5.14E-04	1.30E-02	1.16E-02	6
BP	GO:0035194	posttranscriptional gene silencing by RNA	5.69E-04	1.41E-02	1.26E-02	6
BP	GO:0016441	posttranscriptional gene silencing	5.79E-04	1.41E-02	1.26E-02	6
BP	GO:0006959	humoral immune response	6.84E-04	1.64E-02	1.46E-02	6
BP	GO:1903706	regulation of hemopoiesis	6.97E-04	1.64E-02	1.46E-02	7
BP	GO:0006336	DNA replication-independent nucleosome assembly	7.34E-04	1.69E-02	1.51E-02	3
BP	GO:0034508	centromere complex assembly	7.75E-04	1.73E-02	1.54E-02	3
BP	GO:0034724	DNA replication-independent nucleosome organization	7.75E-04	1.73E-02	1.54E-02	3
BP	GO:0016233	telomere capping	8.18E-04	1.76E-02	1.57E-02	3
BP	GO:0034394	protein localization to cell surface	8.18E-04	1.76E-02	1.57E-02	3
BP	GO:0031047	gene silencing by RNA	8.40E-04	1.78E-02	1.59E-02	6
BP	GO:0043486	histone exchange	9.08E-04	1.89E-02	1.69E-02	3
BP	GO:0060026	convergent extension	1.12E-03	2.29E-02	2.04E-02	2
BP	GO:1903707	negative regulation of hemopoiesis	1.25E-03	2.53E-02	2.26E-02	4
BP	GO:0006303	double-strand break repair via nonhomologous end joining	2.01E-03	3.99E-02	3.56E-02	3
BP	GO:0017148	negative regulation of translation	2.08E-03	4.08E-02	3.64E-02	6
BP	GO:0043044	ATP-dependent chromatin remodeling	2.25E-03	4.34E-02	3.86E-02	3
BP	GO:0034249	negative regulation of cellular amide metabolic process	2.48E-03	4.71E-02	4.20E-02	6
BP	GO:0000726	non-recombinational repair	2.59E-03	4.85E-02	4.33E-02	3
CC	GO:0000786	nucleosome	6.99E-19	9.36E-17	8.31E-17	14
CC	GO:0044815	DNA packaging complex	1.80E-18	1.20E-16	1.07E-16	14
CC	GO:0032993	protein-DNA complex	7.53E-15	3.36E-13	2.99E-13	14
CC	GO:0000788	nuclear nucleosome	5.17E-09	1.73E-07	1.54E-07	6
CC	GO:0000790	nuclear chromatin	2.77E-06	7.42E-05	6.59E-05	9
CC	GO:0000784	nuclear chromosome, telomeric region	6.86E-06	1.53E-04	1.36E-04	6

CC	GO:0000781	chromosome, telomeric region	2.62E-05	5.02E-04	4.45E-04	6
CC	GO:0098687	chromosomal region	2.29E-04	3.83E-03	3.40E-03	7
CC	GO:0005578	proteinaceous extracellular matrix	1.75E-03	2.61E-02	2.31E-02	6
MF	GO:0046982	protein heterodimerization activity	1.89E-10	2.69E-08	2.55E-08	15
MF	GO:0042393	histone binding	8.32E-05	5.91E-03	5.60E-03	6
MF	GO:0031492	nucleosomal DNA binding	5.80E-04	2.48E-02	2.35E-02	3
MF	GO:0033130	acetylcholine receptor binding	6.98E-04	2.48E-02	2.35E-02	2
MF	GO:0031491	nucleosome binding	1.70E-03	4.82E-02	4.57E-02	3
ReactomePA	R-HSA-73728	RNA Polymerase I Promoter Opening	1.65E-21	2.50E-19	1.75E-19	14
ReactomePA	R-HSA-5334118	DNA methylation	2.67E-21	2.50E-19	1.75E-19	14
ReactomePA	R-HSA-5625886	Activated PKN1 stimulates transcription of AR (androgen receptor) regulated genes KLK2 and KLK3	4.26E-21	2.50E-19	1.75E-19	14
ReactomePA	R-HSA-427359	SIRT1 negatively regulates rRNA expression	5.35E-21	2.50E-19	1.75E-19	14
ReactomePA	R-HSA-73777	RNA Polymerase I Chain Elongation	5.40E-21	2.50E-19	1.75E-19	15
ReactomePA	R-HSA-3214815	HDACs deacetylate histones	1.09E-20	4.18E-19	2.93E-19	15
ReactomePA	R-HSA-212300	PRC2 methylates histones and DNA	1.58E-20	5.23E-19	3.67E-19	14
ReactomePA	R-HSA-2299718	Condensation of Prophase Chromosomes	1.95E-20	5.63E-19	3.95E-19	14
ReactomePA	R-HSA-427389	ERCC6 (CSB) and EHMT2 (G9a) positively regulate rRNA expression	2.92E-20	7.50E-19	5.26E-19	14
ReactomePA	R-HSA-427413	NoRC negatively regulates rRNA expression	7.34E-20	1.69E-18	1.19E-18	15
ReactomePA	R-HSA-5250941	Negative epigenetic regulation of rRNA expression	1.14E-19	2.19E-18	1.54E-18	15
ReactomePA	R-HSA-73854	RNA Polymerase I Promoter Clearance	1.14E-19	2.19E-18	1.54E-18	15
ReactomePA	R-HSA-73864	RNA Polymerase I Transcription	1.75E-19	3.11E-18	2.18E-18	15
ReactomePA	R-HSA-912446	Meiotic recombination	2.23E-19	3.68E-18	2.58E-18	14
ReactomePA	R-HSA-201722	Formation of the beta-catenin:TCF transactivating complex	4.36E-19	6.30E-18	4.42E-18	14
ReactomePA	R-HSA-5250924	B-WICH complex positively regulates rRNA expression	4.36E-19	6.30E-18	4.42E-18	14

ReactomePA	R-HSA-5625740	RHO GTPases activate PKNs	8.26E-19	1.12E-17	7.87E-18	14
ReactomePA	R-HSA-8936459	RUNX1 regulates genes involved in megakaryocyte differentiation and platelet function	1.12E-18	1.44E-17	1.01E-17	14
ReactomePA	R-HSA-977225	Amyloid fiber formation	1.76E-18	2.14E-17	1.50E-17	14
ReactomePA	R-HSA-5578749	Transcriptional regulation by small RNAs	3.61E-18	4.17E-17	2.92E-17	14
ReactomePA	R-HSA-5250913	Positive epigenetic regulation of rRNA expression	4.15E-18	4.56E-17	3.20E-17	14
ReactomePA	R-HSA-3214847	HATs acetylate histones	7.05E-18	7.16E-17	5.02E-17	15
ReactomePA	R-HSA-2559582	Senescence-Associated Secretory Phenotype (SASP)	7.13E-18	7.16E-17	5.02E-17	14
ReactomePA	R-HSA-212165	Epigenetic regulation of gene expression	1.20E-17	1.16E-16	8.11E-17	15
ReactomePA	R-HSA-1500620	Meiosis	2.24E-17	2.07E-16	1.45E-16	14
ReactomePA	R-HSA-5617472	Activation of anterior HOX genes in hindbrain development during early embryogenesis	3.21E-17	2.75E-16	1.93E-16	14
ReactomePA	R-HSA-5619507	Activation of HOX genes during differentiation	3.21E-17	2.75E-16	1.93E-16	14
ReactomePA	R-HSA-2559580	Oxidative Stress Induced Senescence	4.56E-17	3.77E-16	2.64E-16	14
ReactomePA	R-HSA-8939236	RUNX1 regulates transcription of genes involved in differentiation of HSCs	8.04E-17	6.40E-16	4.49E-16	14
ReactomePA	R-HSA-171306	Packaging Of Telomere Ends	9.52E-17	7.33E-16	5.15E-16	11
ReactomePA	R-HSA-211000	Gene Silencing by RNA	1.24E-16	9.26E-16	6.50E-16	14
ReactomePA	R-HSA-68875	Mitotic Prophase	2.58E-16	1.86E-15	1.31E-15	14
ReactomePA	R-HSA-1474165	Reproduction	3.85E-16	2.69E-15	1.89E-15	14
ReactomePA	R-HSA-606279	Deposition of new CENPA-containing nucleosomes at the centromere	6.18E-15	4.08E-14	2.86E-14	11
ReactomePA	R-HSA-774815	Nucleosome assembly	6.18E-15	4.08E-14	2.86E-14	11
ReactomePA	R-HSA-201681	TCF dependent signaling in response to WNT	1.27E-14	8.13E-14	5.71E-14	15
ReactomePA	R-HSA-1221632	Meiotic synapsis	1.32E-14	8.23E-14	5.77E-14	11
ReactomePA	R-HSA-2559586	DNA Damage/Telomere Stress Induced Senescence	1.52E-14	9.26E-14	6.50E-14	11

ReactomePA	R-HSA-157579	Telomere Maintenance	2.02E-14	1.20E-13	8.41E-14	11
ReactomePA	R-HSA-2559583	Cellular Senescence	2.52E-14	1.46E-13	1.02E-13	14
ReactomePA	R-HSA-195721	Signaling by WNT	1.16E-13	6.55E-13	4.60E-13	16
ReactomePA	R-HSA-3247509	Chromatin modifying enzymes	1.46E-13	7.86E-13	5.52E-13	15
ReactomePA	R-HSA-4839726	Chromatin organization	1.46E-13	7.86E-13	5.52E-13	15
ReactomePA	R-HSA-8878171	Transcriptional regulation by RUNX1	4.23E-13	2.22E-12	1.56E-12	14
ReactomePA	R-HSA-73886	Chromosome Maintenance	5.68E-13	2.92E-12	2.05E-12	11
ReactomePA	R-HSA-3214858	RMTs methylate histone arginines	6.44E-13	3.24E-12	2.27E-12	10
ReactomePA	R-HSA-195258	RHO GTPase Effectors	1.22E-12	5.98E-12	4.20E-12	15
ReactomePA	R-HSA-68886	M Phase	2.74E-11	1.32E-10	9.25E-11	15
ReactomePA	R-HSA-194315	Signaling by Rho GTPases	1.52E-10	7.18E-10	5.04E-10	15
ReactomePA	R-HSA-5693571	Nonhomologous End-Joining (NHEJ)	3.33E-10	1.54E-09	1.08E-09	8
ReactomePA	R-HSA-5693565	Recruitment and ATM-mediated phosphorylation of repair and signaling proteins at DNA double strand breaks	7.36E-10	3.33E-09	2.34E-09	8
ReactomePA	R-HSA-5693606	DNA Double Strand Break Response	8.18E-10	3.64E-09	2.55E-09	8
ReactomePA	R-HSA-2262752	Cellular responses to stress	1.08E-09	4.70E-09	3.29E-09	14
ReactomePA	R-HSA-69473	G2/M DNA damage checkpoint	4.47E-09	1.91E-08	1.34E-08	8
ReactomePA	R-HSA-5693607	Processing of DNA double-strand break ends	5.74E-09	2.41E-08	1.69E-08	8
ReactomePA	R-HSA-3214842	HDMs demethylate histones	5.90E-08	2.44E-07	1.71E-07	6
ReactomePA	R-HSA-5693567	HDR through Homologous Recombination (HR) or Single Strand Annealing (SSA)	6.05E-08	2.45E-07	1.72E-07	8
ReactomePA	R-HSA-5693538	Homology Directed Repair	8.56E-08	3.41E-07	2.39E-07	8
ReactomePA	R-HSA-5689880	Ub-specific processing proteases	2.45E-07	9.60E-07	6.74E-07	9
ReactomePA	R-HSA-5693532	DNA Double-Strand Break Repair	3.57E-07	1.37E-06	9.64E-07	8
ReactomePA	R-HSA-69481	G2/M Checkpoints	3.91E-07	1.48E-06	1.04E-06	8
ReactomePA	R-HSA-3214841	PKMTs methylate histone lysines	4.41E-07	1.64E-06	1.15E-06	6

ReactomePA	R-HSA-69620	Cell Cycle Checkpoints	2.70E-06	9.90E-06	6.94E-06	9
ReactomePA	R-HSA-5688426	Deubiquitination	3.02E-06	1.09E-05	7.64E-06	9
ReactomePA	R-HSA-8866654	E3 ubiquitin ligases ubiquitinate target proteins	3.62E-06	1.29E-05	9.03E-06	5
ReactomePA	R-HSA-73894	DNA Repair	5.01E-06	1.75E-05	1.23E-05	9
ReactomePA	R-HSA-5689901	Metalloprotease DUBs	1.59E-05	5.46E-05	3.83E-05	4
ReactomePA	R-HSA-8852135	Protein ubiquitination	1.61E-05	5.46E-05	3.83E-05	5
ReactomePA	R-HSA-5689603	UCH proteinases	8.42E-04	2.82E-03	1.98E-03	4
ReactomePA	R-HSA-1266695	Interleukin-7 signaling	9.16E-04	3.02E-03	2.12E-03	3
ReactomePA	R-HSA-4551638	SUMOylation of chromatin organization proteins	3.08E-03	1.00E-02	7.03E-03	3
ReactomePA	R-HSA-3108232	SUMO E3 ligases SUMOylate target proteins	1.52E-02	4.89E-02	3.43E-02	3

BP: biological process; CC: cellular component; MF: molecular function

Supplementary Table 8. Functional enrichment analysis of differentially expressed genes identified by RankCompV2.

Category	ID	Description	*P*	p.adjust	qvalue	Count
BP	GO:0006334	nucleosome assembly	4.07E-14	5.07E-11	4.64E-11	13
BP	GO:0031497	chromatin assembly	2.00E-13	1.25E-10	1.14E-10	13
BP	GO:0034728	nucleosome organization	4.28E-13	1.78E-10	1.63E-10	13
BP	GO:0006333	chromatin assembly or disassembly	1.23E-12	3.83E-10	3.50E-10	13
BP	GO:0065004	protein-DNA complex assembly	1.53E-12	3.83E-10	3.50E-10	14
BP	GO:0006323	DNA packaging	3.91E-12	8.12E-10	7.43E-10	13
BP	GO:0071824	protein-DNA complex subunit organization	7.84E-12	1.40E-09	1.28E-09	14
BP	GO:0071103	DNA conformation change	2.50E-10	3.90E-08	3.57E-08	13
BP	GO:0002227	innate immune response in mucosa	3.04E-08	4.21E-06	3.85E-06	5
BP	GO:0006342	chromatin silencing	3.68E-08	4.59E-06	4.20E-06	8
BP	GO:0045814	negative regulation of gene expression, epigenetic	9.94E-08	1.13E-05	1.03E-05	8
BP	GO:0002385	mucosal immune response	2.25E-07	2.33E-05	2.14E-05	5
BP	GO:0002251	organ or tissue specific immune response	3.08E-07	2.96E-05	2.71E-05	5
BP	GO:0019731	antibacterial humoral response	1.16E-06	1.04E-04	9.49E-05	5
BP	GO:0050830	defense response to Gram-positive bacterium	2.43E-06	2.02E-04	1.85E-04	6
BP	GO:0061844	antimicrobial humoral immune response mediated by antimicrobial peptide	3.28E-06	2.56E-04	2.34E-04	5
BP	GO:0019730	antimicrobial humoral response	5.59E-06	4.10E-04	3.75E-04	6
BP	GO:0006335	DNA replication-dependent nucleosome assembly	9.95E-06	6.53E-04	5.97E-04	4
BP	GO:0034723	DNA replication-dependent nucleosome organization	9.95E-06	6.53E-04	5.97E-04	4
BP	GO:0000183	chromatin silencing at rDNA	2.00E-05	1.25E-03	1.14E-03	4
BP	GO:0051290	protein heterotetramerization	3.61E-05	2.14E-03	1.96E-03	4
BP	GO:1902036	regulation of hematopoietic stem cell differentiation	7.72E-05	4.38E-03	4.00E-03	5
BP	GO:0016458	gene silencing	8.35E-05	4.53E-03	4.14E-03	9

BP	GO:0060218	hematopoietic stem cell differentiation	1.20E-04	5.97E-03	5.47E-03	5
BP	GO:1901532	regulation of hematopoietic progenitor cell differentiation	1.20E-04	5.97E-03	5.47E-03	5
BP	GO:0042742	defense response to bacterium	2.32E-04	1.11E-02	1.02E-02	7
BP	GO:0045652	regulation of megakaryocyte differentiation	3.08E-04	1.42E-02	1.30E-02	4
BP	GO:2000736	regulation of stem cell differentiation	3.36E-04	1.50E-02	1.37E-02	5
BP	GO:0045815	positive regulation of gene expression, epigenetic	3.75E-04	1.61E-02	1.48E-02	4
BP	GO:0030219	megakaryocyte differentiation	6.64E-04	2.76E-02	2.52E-02	4
BP	GO:0022600	digestive system process	6.91E-04	2.78E-02	2.54E-02	4
BP	GO:0007586	digestion	7.48E-04	2.83E-02	2.59E-02	5
BP	GO:0032200	telomere organization	7.48E-04	2.83E-02	2.59E-02	5
BP	GO:0040029	regulation of gene expression, epigenetic	1.03E-03	3.77E-02	3.45E-02	8
BP	GO:0060964	regulation of gene silencing by miRNA	1.08E-03	3.86E-02	3.53E-02	4
BP	GO:0060147	regulation of posttranscriptional gene silencing	1.20E-03	4.05E-02	3.71E-02	4
BP	GO:0060966	regulation of gene silencing by RNA	1.20E-03	4.05E-02	3.71E-02	4
BP	GO:0002244	hematopoietic progenitor cell differentiation	1.24E-03	4.06E-02	3.71E-02	5
CC	GO:0000786	nucleosome	3.81E-23	5.68E-21	5.10E-21	17
CC	GO:0044815	DNA packaging complex	1.22E-22	9.12E-21	8.19E-21	17
CC	GO:0032993	protein-DNA complex	3.49E-18	1.74E-16	1.56E-16	17
CC	GO:0000790	nuclear chromatin	9.26E-06	3.45E-04	3.09E-04	9
CC	GO:0000788	nuclear nucleosome	2.03E-05	6.06E-04	5.44E-04	4
CC	GO:0000781	chromosome, telomeric region	5.93E-05	1.47E-03	1.32E-03	6
CC	GO:0000784	nuclear chromosome, telomeric region	1.98E-04	4.21E-03	3.78E-03	5
MF	GO:0046982	protein heterodimerization activity	2.45E-12	3.97E-10	3.71E-10	18
ReactomePA	R-HSA-3214815	HDACs deacetylate histones	1.37E-20	5.17E-18	4.14E-18	16
ReactomePA	R-HSA-3214847	HATs acetylate histones	3.90E-19	7.36E-17	5.90E-17	17
ReactomePA	R-HSA-73728	RNA Polymerase I Promoter Opening	5.35E-18	6.73E-16	5.39E-16	13

ReactomePA	R-HSA-5334118	DNA methylation	8.33E-18	7.85E-16	6.29E-16	13
ReactomePA	R-HSA-5625886	Activated PKN1 stimulates transcription of AR (androgen receptor) regulated genes KLK2 and KLK3	1.28E-17	9.63E-16	7.72E-16	13
ReactomePA	R-HSA-427359	SIRT1 negatively regulates rRNA expression	1.57E-17	9.89E-16	7.92E-16	13
ReactomePA	R-HSA-212300	PRC2 methylates histones and DNA	4.25E-17	2.29E-15	1.83E-15	13
ReactomePA	R-HSA-2299718	Condensation of Prophase Chromosomes	5.13E-17	2.42E-15	1.94E-15	13
ReactomePA	R-HSA-427389	ERCC6 (CSB) and EHMT2 (G9a) positively regulate rRNA expression	7.43E-17	3.11E-15	2.49E-15	13
ReactomePA	R-HSA-912446	Meiotic recombination	4.77E-16	1.80E-14	1.44E-14	13
ReactomePA	R-HSA-73777	RNA Polymerase I Chain Elongation	7.58E-16	2.55E-14	2.05E-14	13
ReactomePA	R-HSA-201722	Formation of the beta-catenin:TCF transactivating complex	8.81E-16	2.55E-14	2.05E-14	13
ReactomePA	R-HSA-5250924	B-WICH complex positively regulates rRNA expression	8.81E-16	2.55E-14	2.05E-14	13
ReactomePA	R-HSA-5625740	RHO GTPases activate PKNs	1.58E-15	3.87E-14	3.10E-14	13
ReactomePA	R-HSA-3247509	Chromatin modifying enzymes	1.64E-15	3.87E-14	3.10E-14	18
ReactomePA	R-HSA-4839726	Chromatin organization	1.64E-15	3.87E-14	3.10E-14	18
ReactomePA	R-HSA-171306	Packaging Of Telomere Ends	1.77E-15	3.93E-14	3.15E-14	11
ReactomePA	R-HSA-2559580	Oxidative Stress Induced Senescence	2.05E-15	4.15E-14	3.33E-14	14
ReactomePA	R-HSA-8936459	RUNX1 regulates genes involved in megakaryocyte differentiation and platelet function	2.09E-15	4.15E-14	3.33E-14	13
ReactomePA	R-HSA-977225	Amyloid fiber formation	3.16E-15	5.95E-14	4.77E-14	13
ReactomePA	R-HSA-8939236	RUNX1 regulates transcription of genes involved in differentiation of HSCs	3.58E-15	6.43E-14	5.15E-14	14
ReactomePA	R-HSA-5578749	Transcriptional regulation by small RNAs	6.08E-15	1.04E-13	8.34E-14	13
ReactomePA	R-HSA-2559586	DNA Damage/Telomere Stress Induced Senescence	6.83E-15	1.04E-13	8.34E-14	12
ReactomePA	R-HSA-427413	NoRC negatively regulates rRNA expression	6.90E-15	1.04E-13	8.34E-14	13
ReactomePA	R-HSA-5250913	Positive epigenetic regulation of rRNA expression	6.90E-15	1.04E-13	8.34E-14	13

ReactomePA	R-HSA-5250941	Negative epigenetic regulation of rRNA expression	1.00E-14	1.40E-13	1.12E-13	13
ReactomePA	R-HSA-73854	RNA Polymerase I Promoter Clearance	1.00E-14	1.40E-13	1.12E-13	13
ReactomePA	R-HSA-2559582	Senescence-Associated Secretory Phenotype (SASP)	1.13E-14	1.52E-13	1.22E-13	13
ReactomePA	R-HSA-73864	RNA Polymerase I Transcription	1.44E-14	1.87E-13	1.50E-13	13
ReactomePA	R-HSA-1500620	Meiosis	3.22E-14	4.05E-13	3.24E-13	13
ReactomePA	R-HSA-5617472	Activation of anterior HOX genes in hindbrain development during early embryogenesis	4.48E-14	5.28E-13	4.23E-13	13
ReactomePA	R-HSA-5619507	Activation of HOX genes during differentiation	4.48E-14	5.28E-13	4.23E-13	13
ReactomePA	R-HSA-2559583	Cellular Senescence	5.14E-14	5.88E-13	4.71E-13	15
ReactomePA	R-HSA-606279	Deposition of new CENPA-containing nucleosomes at the centromere	1.12E-13	1.21E-12	9.70E-13	11
ReactomePA	R-HSA-774815	Nucleosome assembly	1.12E-13	1.21E-12	9.70E-13	11
ReactomePA	R-HSA-211000	Gene Silencing by RNA	1.54E-13	1.61E-12	1.29E-12	13
ReactomePA	R-HSA-1221632	Meiotic synapsis	2.38E-13	2.43E-12	1.94E-12	11
ReactomePA	R-HSA-68875	Mitotic Prophase	3.00E-13	2.97E-12	2.38E-12	13
ReactomePA	R-HSA-157579	Telomere Maintenance	3.65E-13	3.53E-12	2.83E-12	11
ReactomePA	R-HSA-1474165	Reproduction	4.31E-13	4.07E-12	3.26E-12	13
ReactomePA	R-HSA-212165	Epigenetic regulation of gene expression	5.16E-13	4.74E-12	3.80E-12	13
ReactomePA	R-HSA-8878171	Transcriptional regulation by RUNX1	1.02E-12	9.20E-12	7.37E-12	15
ReactomePA	R-HSA-73886	Chromosome Maintenance	9.94E-12	8.71E-11	6.98E-11	11
ReactomePA	R-HSA-201681	TCF dependent signaling in response to WNT	1.19E-11	1.02E-10	8.16E-11	14
ReactomePA	R-HSA-5693571	Nonhomologous End-Joining (NHEJ)	7.82E-11	6.55E-10	5.25E-10	9
ReactomePA	R-HSA-5689880	Ub-specific processing proteases	8.85E-11	7.25E-10	5.81E-10	13
ReactomePA	R-HSA-195721	Signaling by WNT	1.04E-10	8.38E-10	6.72E-10	15
ReactomePA	R-HSA-5693565	Recruitment and ATM-mediated phosphorylation of repair and signaling proteins at DNA double strand breaks	1.91E-10	1.50E-09	1.20E-09	9

ReactomePA	R-HSA-5693606	DNA Double Strand Break Response	2.16E-10	1.66E-09	1.33E-09	9
ReactomePA	R-HSA-2262752	Cellular responses to stress	4.15E-10	3.13E-09	2.51E-09	16
ReactomePA	R-HSA-69473	G2/M DNA damage checkpoint	1.46E-09	1.08E-08	8.65E-09	9
ReactomePA	R-HSA-5693607	Processing of DNA double-strand break ends	1.93E-09	1.40E-08	1.12E-08	9
ReactomePA	R-HSA-5688426	Deubiquitination	3.57E-09	2.54E-08	2.04E-08	13
ReactomePA	R-HSA-3214858	RMTs methylate histone arginines	7.52E-09	5.25E-08	4.21E-08	8
ReactomePA	R-HSA-195258	RHO GTPase Effectors	8.14E-09	5.58E-08	4.47E-08	13
ReactomePA	R-HSA-68886	M Phase	1.22E-08	8.23E-08	6.59E-08	14
ReactomePA	R-HSA-69481	G2/M Checkpoints	1.57E-08	1.04E-07	8.32E-08	10
ReactomePA	R-HSA-5693567	HDR through Homologous Recombination (HR) or Single Strand Annealing (SSA)	2.71E-08	1.76E-07	1.41E-07	9
ReactomePA	R-HSA-5693538	Homology Directed Repair	4.00E-08	2.55E-07	2.05E-07	9
ReactomePA	R-HSA-5693532	DNA Double-Strand Break Repair	1.97E-07	1.24E-06	9.92E-07	9
ReactomePA	R-HSA-194315	Signaling by Rho GTPases	4.31E-07	2.67E-06	2.14E-06	13
ReactomePA	R-HSA-8866654	E3 ubiquitin ligases ubiquitinate target proteins	5.11E-07	3.11E-06	2.49E-06	6
ReactomePA	R-HSA-69620	Cell Cycle Checkpoints	2.78E-06	1.66E-05	1.33E-05	10
ReactomePA	R-HSA-8852135	Protein ubiquitination	3.08E-06	1.81E-05	1.45E-05	6
ReactomePA	R-HSA-3214842	HDMs demethylate histones	6.97E-06	4.04E-05	3.24E-05	5
ReactomePA	R-HSA-73894	DNA Repair	3.90E-05	2.23E-04	1.79E-04	9
ReactomePA	R-HSA-5689901	Metalloprotease DUBs	4.18E-05	2.35E-04	1.89E-04	4
ReactomePA	R-HSA-5689603	UCH proteinases	2.01E-04	1.11E-03	8.93E-04	5
ReactomePA	R-HSA-3214841	PKMTs methylate histone lysines	5.38E-04	2.94E-03	2.36E-03	4

BP: biological process; CC: cellular component; MF: molecular function
